# Mental Health Admissions to Paediatric Wards Study (MAPS): protocol of a prospective study of mental health admissions to paediatric wards in England using surveillance and qualitative methods

**DOI:** 10.1136/bmjpo-2023-002186

**Published:** 2024-01-25

**Authors:** Lee Duncan Hudson, Adriana Vázquez-Vázquez, Faith Gibson, Kirsty Phillips, Gabrielle Mathews, Helen Roberts, Francesca Cornaglia, Damian Roland, Joseph Ward, Dasha E Nicholls, Holly Elphinstone, Russell Viner

**Affiliations:** 1 University College London Great Ormond Street Institute of Child Health, London, UK; 2 Population, Policy and Practice, University College London Great Ormond Street Institute of Child Health, London, UK; 3 Great Ormond Street Hospital for Children NHS Trust, London, UK; 4 CYP Transformation Team, NHS England and NHS Improvement London, London, UK; 5 Queen Mary University of London, London, UK; 6 SAPPHIRE Group, Population Health Sciences, Leicester University, Leicester, UK; 7 Paediatric Emergency Medicine Leicester Academic (PEMLA) Group, Children's Emergency Department, Leicester Royal Infirmary, Leicester, UK; 8 Division of Psychiatry, Imperial College London, London, UK

**Keywords:** Adolescent Health, Child Psychiatry, Data Collection, Epidemiology, Qualitative research

## Abstract

**Introduction:**

Children and young people (CYP) presenting with a mental health (MH) crisis are frequently admitted to general acute paediatric wards as a place of safety. Prior to the pandemic, a survey in England showed that CYP occupied 6% of general paediatric inpatient beds due to an MH crisis, and there have been longstanding concerns about the quality of care to support these patients in this setting. MAPS aims to generate a Theory of Change (ToC) model to improve the quality of care for CYP admitted to acute paediatric services after presenting with an MH crisis. Here, we describe work packages (WPs) 2 and 3 of the study, which have been granted ethics approval.

**Methods and analysis:**

We will undertake a national (England), sequential, mixed-methods study to inform a ToC framework alongside a stakeholder group consisting of patients, families/carers and healthcare professionals (HCPs). Our study consists of four WPs undertaken over 30 months. WP2 is limited to working with stakeholders to develop a data collection instrument and then use this in a prospective study of MH admissions over 6 months in 15 purposively recruited acute paediatric wards across England. WP3 consists of gathering the views of CYP, their families/carers and HCPs during admissions using semistructured interviews.

**Ethics and dissemination:**

WP2 and WP3 received ethical approval (ref: 23/LO/0349). We will publish the overall synthesis of data and the final ToC to improve care of CYP with MH crisis admitted to general acute paediatric settings. As co-producers of the ToC, we will work with our stakeholder group to ensure wide dissemination of findings. Potential impacts will be upon service development, new models of care, training and workforce planning.

**PROSPERO registration number:**

CRD42022350655.

**WHAT IS ALREADY KNOWN ON THIS TOPIC**

- There is evidence that both the number of paediatric admissions and the severity of MH crisis in CYP have increased.
- Children’s wards are not designed to treat unwell CYP with MH problems and sometimes HCPs working on them haven’t had enough training.
- HCPs from children’s wards are reporting that they are finding supporting CYP admitted with MH problems challenging.

**WHAT THIS STUDY ADDS**

- This study will characterise admissions in terms of sociodemographic factors, diagnoses and factors influencing decisions to admit CYP to paediatric wards for primary MH problems.
- This study will describe the views and experiences of CYP, families/carers and HCPs during MH admissions to paediatric wards.
- This study will generate a ToC model to positively impact the quality of care for CYP admitted to paediatric services because of a MH crisis.

**HOW THIS STUDY MIGHT AFFECT RESEARCH, PRACTICE OR POLICY**

- By producing a ToC approach, we expect to generate a system map to identify transformation plans to share with policymakers, commissioners, service leads and professionals.
- Our data and outputs will enable advocating for and improving cultural views on CYP with MH crises as part of the acute paediatric system.

## Introduction

Children and young people (CYP) presenting with a mental health (MH) crisis are frequently admitted to general acute paediatric wards as a place of safety, despite not always having the resources or training.^1 2^ Before the pandemic, a survey carried out in 2019 with 60% of acute paediatric services in England found that 6% of general paediatric beds were occupied by CYP with MH problems.^3^ Moreover, data from London suggest that the management of CYP with MH problems was one of the main challenges for acute children’s services.^3^

The rise in MH problems among CYP during the COVID-19 pandemic has been also well described.^4 5^ Recent national data report that being at high risk of MH problems rose from one in nine in 2017 to one in six by 2021, with a doubling of the proportion of CYP at risk of eating problems over that same period.^6^ During the first wave, acute services became ‘default providers’ where community or inpatient Child and Adolescent Mental Health Services (CAMHS) were not accessible and during the third wave, admissions to acute wards appeared to peak.^7^ This mismatch of greater distress and reduced access led to increases in already unmet needs.^8^

**Figure 1:**
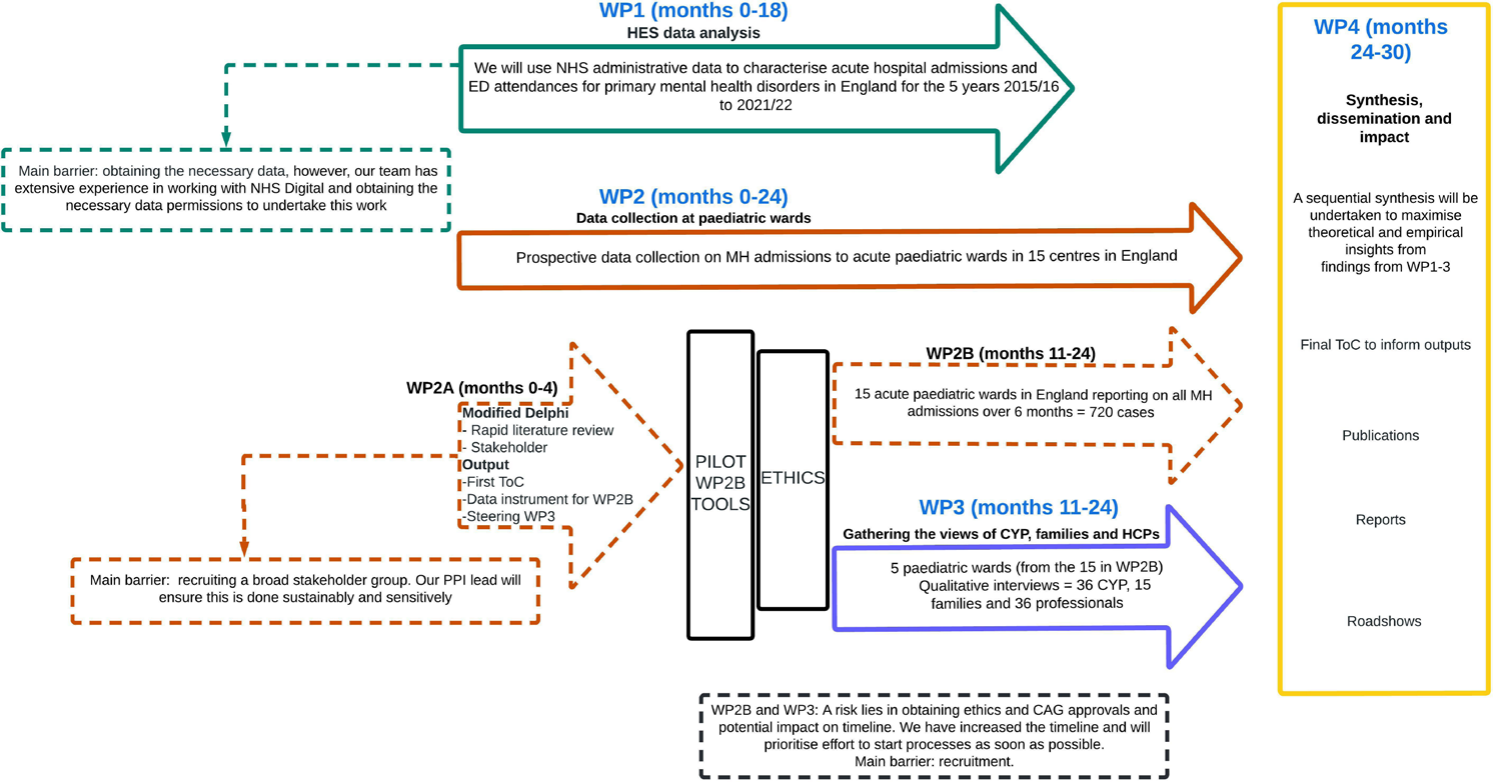
MAPS work package (WP) flow diagram. CAG, Confidentiality Advisory Group; CYP, children and young people; ED, emergency department; HCPs, healthcare professionals; HES, Hospital Episode Statistics; MH, mental health; NHS, National Health Service; PPI, patient and public involvement; ToC, Theory of Change.

Although amplified by the pandemic, MH admissions to acute paediatric wards are a longstanding issue that has been identified as a leading safety and quality concern for acute paediatric providers for some years.^1 3^ The MAPS Study aims to generate a Theory of Change (ToC) model to improve the quality of care for CYP admitted to acute paediatric services after presenting with an MH crisis. Our study consists of four work packages (WPs). Here, we describe WP2 and WP3 (figure 1), which have been granted ethics approval. WP1, limited to using national routine administrative data to identify and characterise trends in MH admissions in acute paediatric wards in England between 2015 and 2022, is currently undergoing ethics assessment, and the protocol will be described separately when ethics processes are complete. For WP4, we will synthesise, with our stakeholder group, the data delivered by WP1–3 to create a ToC model for agreed impacts to inform service provision, potentially including the development of new pathways or models of care needed to improve the care of CYP admitted to acute wards. Overall, MAPS will look in detail a serious healthcare problem for how CYP are treated and have access to and care for their MH. It will impact positively by providing the information needed to develop the way care is provided for CYP and families/carers.

## Methods and analysis

We will use a ToC approach as our framework, which uses logic (quantitative) data and co-production (qualitative) data to map change. This approach has been applied to a range of areas of health and social care improvement settings.^9–11^

### Study design

We will undertake a national, sequential, mixed-methods study to inform a ToC framework alongside a stakeholder group consisting of patients, families/carers and healthcare professionals (HCPs). Three WPs will deliver the types of evidence needed to inform our ToC. Here, we describe the study designs for WP2 and WP3.

WP2, consisting of WP2A and WP2B, aims to investigate factors influencing decisions to admit CYP to paediatric wards for primary MH problems, including why possible alternative services were not used, and to characterise the care given, treatment outcomes and subsequent service use. To achieve this aim, a detailed prospective data collection on MH admissions to acute paediatric wards in 15 centres in England will be carried out.

WP3 aims to explore the views and experiences of CYP admitted and the views and experiences of their families/carers and HCPs, concerning their admission, care and treatment. To fulfil this aim, we will (1) explore CYP’s and their families/carers’ experience of their admission to a paediatric ward, including an understanding of the reasons for admission, the care and treatment received before and during this admission; (2) explore HCPs’ experience of caring for CYP with primary MH problems and their families/carers, to understand more fully their preparation for this role, as well as enablers and barriers to delivery of individualised patient-centred care; and (3) determine recommendations for the improvement of care, treatment and outcomes of CYP and their families/carers.

### WP2: development of data collection instrument (WP2A) and data collection at paediatric wards (WP2B)

We used a modified Delphi approach, to work with our stakeholder group in the co-creation of our detailed prospective data collection tool (WP2A). Members of our stakeholder group and the patient and public involvement and engagement (PPIE) group were the experts in our Delphi.

First, our literature review informed an initial set of key domains, which were modified after consultation with the group. Our review aimed to systematically examine the evidence on CYP admitted to children’s/paediatric wards because of a primary MH reason, mainly in MH crises. We addressed five search questions to inform about trends and/or the number of admissions, the risk factors for adverse care, the experiences of CYP, families/carers and staff and the evidence of projects aimed at improving the care during admissions. Searches were carried out in PubMed, Embase, PsycINFO, Web of Science and Google Scholar and were restricted to the years 1990–2023. The protocol for this review was registered with PROSPERO (CRD42022350655). The searches located 10 826 articles and 8332 studies were left after duplicate removal. Ninety-two studies were retrieved for full-text assessment and 61 were excluded based on this assessment. Thirty-one studies are included in this review.

Results allowed us to answer four of our five search questions and studies could be included under more than one review question. Seventeen studies provided information about trends in admissions and highlighted that admissions are increasing and are a longstanding issue.^7 12–27^ Thirteen studies provided data about the views/experiences of HCPs.^7 12 28–38^ Overall, HCPs highlighted concerns around the appropriateness of ward environments and the training and skills to manage CYP with MH diagnoses. We only found two studies that provided data about the experiences of CYP during admission.^32 39^ One of the studies reported that CYP expressed appreciation for compassionate clinicians and for receiving information about what to expect during their hospital stay. The other study reported that CYP recalled many emotions during admission, including fear, anger, depression and confusion, about why they were being admitted. Finally, we only found four studies aimed at improving the care of CYP during admissions, which focused on improving the adequate management of patients through the promotion of communication skills, the development of a joint working model with CAMHS, and the improvement of professional competence and training.^19 35 40 41^

Then, we developed the tool and sent it for comments/suggestions from the group. We incorporated the changes, and we built the online web tool form to enable data entry. We used the REDCap system in which data are directly imported into the secure University College London Data Safe Haven. For round two, we tested the instrument and online web form in three sites using a series of ‘dummy’ or fictitious patients. Feedback from this piloting was incorporated into the final instrument. Table 1 shows the section headings and examples of questions that compose each section of the tool.

**Table 1:** MAPS tool

Section headings	Questions (examples)
Key facts about admission	Date of admission and discharge Patient characteristics (age, sex, ethnicity)
Further detail about management during the admission	Would you consider this admission as planned or unplanned? Did the patient receive restraint during the admission?
Mental health assessment/support during admission	Was the patient seen by a mental health professional on the ward? Was guidance given to the inpatient team by a mental health professional during the admission?
Social aspects of the admission	Was the patient known to a local authority social care team prior to admission? Were social work or safeguarding issues raised during the admission?
Information about the lead-up prior to admission	Presentation to ED in the previous 3 months Did the young person initially present alone?
Discharge	Where was the patient discharged to? Did they get planned medical (paediatric follow-up) at discharge?
Overall perceptions of the admission (Likert scales with response options listed in ascending order (strongly disagree to strongly agree))	The young person was involved in the decisions about their care during the admission. The ward was prepared for this admission.

ED, emergency department.

Using the tool, we will prospectively collect data on all primary MH admissions to 15 acute paediatric sites in England over 6 months (WP2B). We will purposively select these sites from children’s hospitals and district general hospitals across different geographical regions, urban and rural, in England. A nominated paediatrician from each site will report data on all CYP admitted who meet the case definition over 6 months. Cases of interest will essentially be any CYP admitted to the acute paediatric ward for a primary MH crisis during the data collection period (table 2).

**Table 2:** Inclusion criteria for MAPS WP2 and WP3

Inclusion criteria	Paediatric sites	CYP	Family/carers/friends	HCPs
WP2	We have already obtained outline agreements from approximately 30 sites that would welcome participation in this project. The purposively selected 15 sites will come from these 30 sites that have already signalled interest. Site in which local NHS services opt out processes are active.	Any CYP (≤18 years of age) admitted to the site for a primary MH crisis during the data collection period (6 months)		
WP3	Five acute paediatric sites will be selected from the 15 anonymised sites involved with WP2.	CYP aged 10–17 years (up to 18th birthday) who live in England CYP admitted to one of the five sites with a primary MH diagnosis	Family members, carers/guardians or friends of CYP, who are 16 years and above, live in England and have been identified by CYP as having played an important role in their care and admission*.	HCPs who are a member of the treating team for CYP working on one of the five selected paediatric wards. HCPs self-identified by CYP as having played an important role in their care during their admission.

*CYP can be included without identifying any family/carers for recruitment to the study.

CYP, children and young people; HCPs, healthcare professionals; MH, mental health; NHS, National Health Service; WP, work package.

In terms of sample size, our survey of 36 sites in January–March 2021 showed a median number of primary MH admissions per centre of 13 per month.^7^ We anticipate there will be a lower number post-pandemic of 8 patients per month across 15 sites, totalling 720 patients. We estimate that data will be collected on 90% of these, providing a sample of 650 for the study. We have not undertaken a formal power calculation but note that 650 patients provide a precision (95% CI) of ±2.3% for a proportion of 10% and ±3.5% for a proportion of 30% for the primary outcomes noted above (given school-age population approximately 8 million).

**Figure 2:**
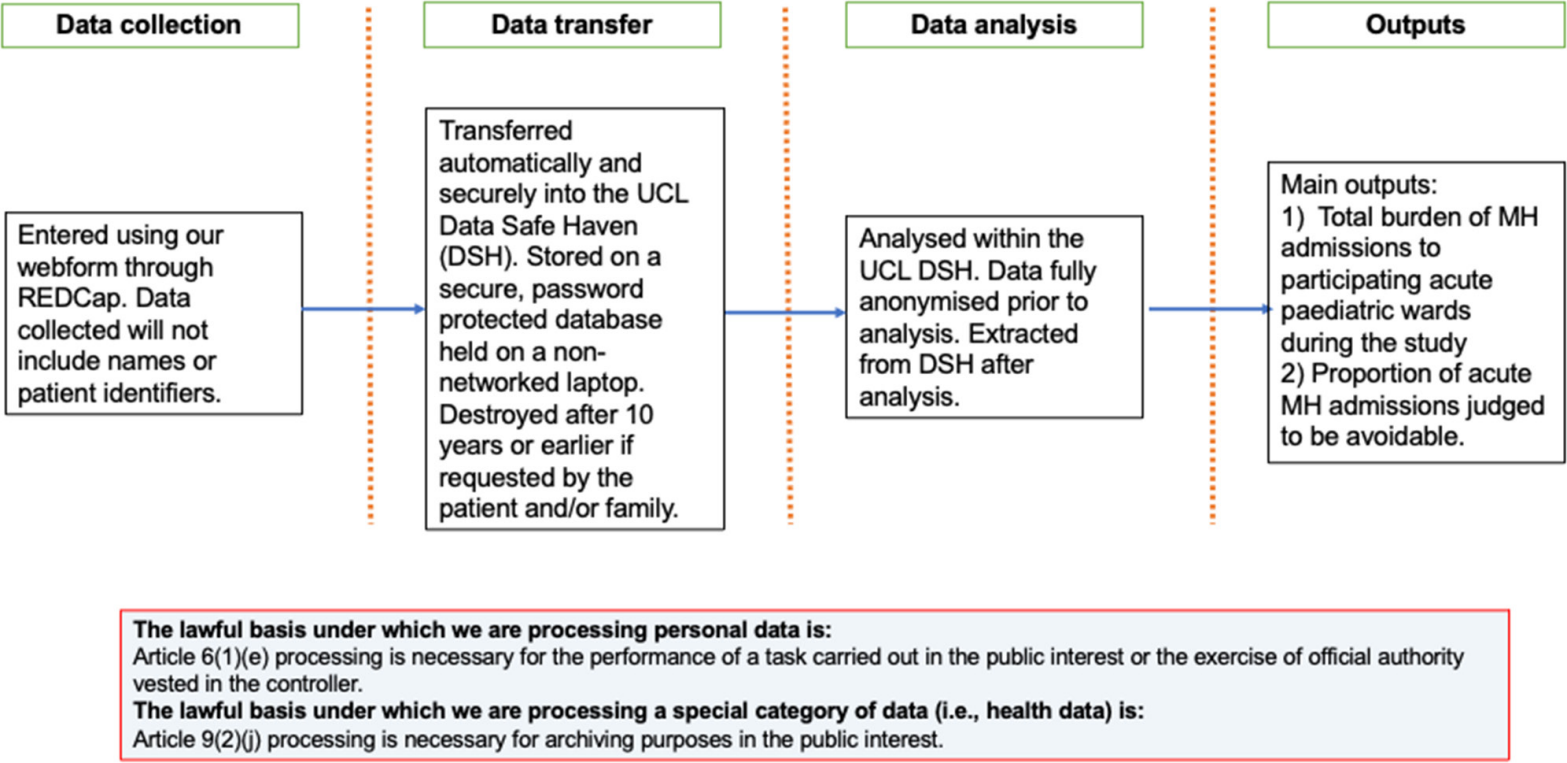
WP2 data flow diagram. MH, mental health; UCL, University College London; WP, work package.

A summary of the WP2 procedure can be seen in figure 2. Reporting of outcomes will be primarily descriptive, and we will minimise formal statistical testing.

### WP3: gathering the views of CYP, families/carers and HCPs

Understanding the experiences of CYP and their families/carers is fundamental to assessing the context of care. The National Health Service England-funded Amplified programme is providing evidence of the importance of and approaches to engaging with CYP, such as supporting and building participation to ensure CYP are involved in decision-making about their care and providing feedback on their experience.^42^ For CYP and families/carers experiencing vulnerabilities, we need to find ways of designing and promoting MH support that works for them. This requires understanding their experiences of care and service provision.

A multiple-case study will be our focus. Five acute paediatric sites will be selected from the 15 sites involved with WP2, with each site representing a case. We will apply a ‘diverse’ case selection approach, which aims to ensure maximum variance of cases along relevant dimensions or criteria.^43 44^ Hence, the five sites selected will provide a rich and diverse sample of CYP experiencing MH problems, as well as family/carers and HCPs from varying disciplines.

**Figure 3:**
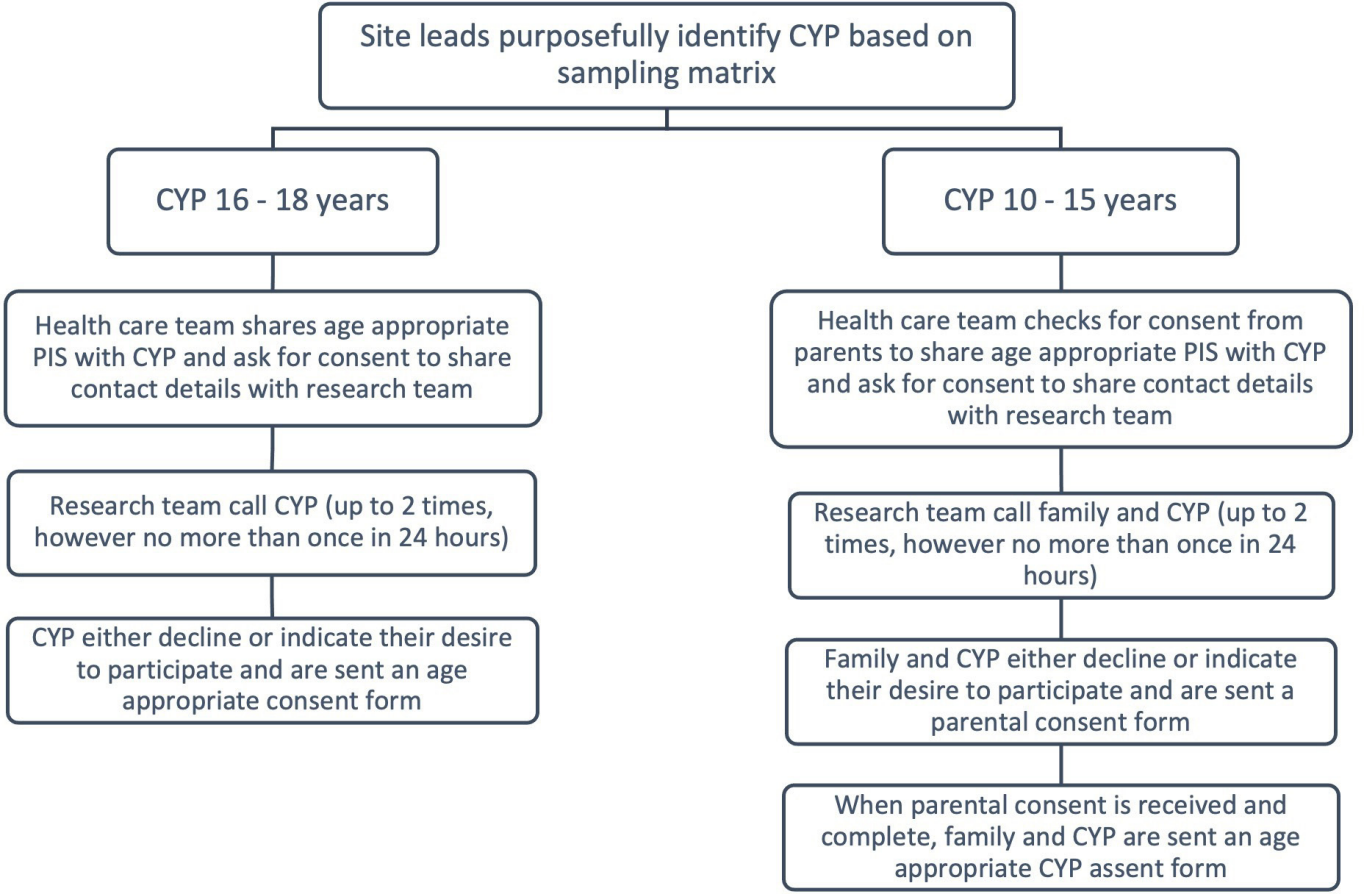
Recruitment flow chart for CYP. CYP, children and young people; PIS, Patient Information Sheet.

Figure 3 illustrates the flow of steps which will be taken to identify and recruit CYP and table 2 the criteria for inclusion. A named staff lead for each of the five sites will support the identification of CYP to be approached. If consent to be contacted is given, a member of the research team would contact the CYP and parents/guardians of the CYP by phone to provide more information and gauge interest in the study. CYP will be asked to identify one to two family members/carers, as well as one to two HCPs from their paediatric centre to participate in data collection. The staff lead for each of the five sites will be asked to facilitate the recruitment of HCPs. For each case, we aim to recruit up to seven to eight CYP (total n=35–40), eight to nine family members/carers (total n=40–45) and four to five HCPs (total n=20–25). However, data collection will continue until a theory has emerged and the data set provides sufficient similarities and contrasts to the emerging theory.^45^

Semistructured interviews will be carried out and will be held virtually (MS Teams/Zoom) or by telephone. Interviews will allow for key questions to be explored with participants (table 3) and for a more in-depth exploration of experiences of care and service provision. Interview schedules and the final version of the key questions that will guide the interviews will be shared with our stakeholder group and patient and public involvement members for discussion before starting data collection.

**Table 3:** Semistructured interviews

Participants	Questions (examples)
CYP	Tell us a little bit about yourself. Can you tell us a bit about your illness? Tell us about your admission into the hospital. What was that like?
Family/carers/friends	Tell us a little bit about yourself. What can you tell us about their admission into the hospital? and their stay on the ward? What was that like do you think?
HCPs	What is your role in caring for young people who have a mental health diagnosis? Tell me about your experiences of this. What about your training for this role, what does that look like? How long have you been doing this role?

CYP, children and young people; HCPs, healthcare professionals.

Our approach will be iterative-inductive analysis as there will be simultaneous sampling with collection and analysis of data, each informing the other. This will allow for structured and defensible flexibility in our study and maximise our ability to respond to theoretical sensitivity.

**Figure 4:**
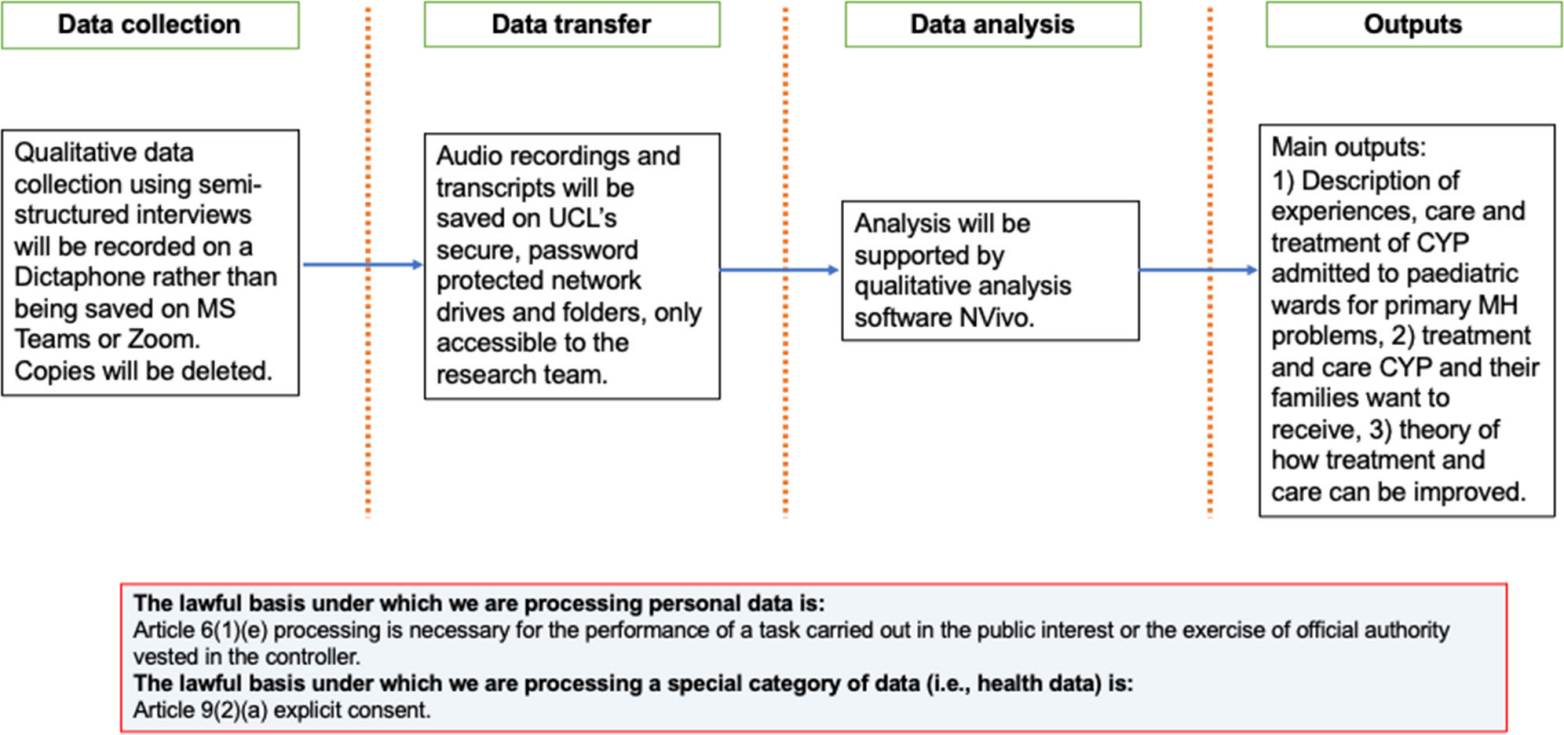
WP3 data flow diagram. CYP, children and young people; MH, mental health; UCL, University College London; WP, work package.

A summary of our study procedure for WP3 can be seen in figure 4.

### Patient and public involvement

We presented our research proposal to members of WP2, to members of the Think4Brum, which is the youth advisory group for Forward Thinking Birmingham, and the GOSH Young Persons’ Advisory Group for research as part of a PPIE initiative. Focus groups of 40 young people (aged <18 years) and parents were held to discuss the acceptability of the methods and the use of data without consent. We received feedback on the importance of the project and the acceptability of collecting the data without consent.

### Dissemination

Regarding dissemination, see WP4 of the study in figure 1. As co-producers of the ToC, we will work with our stakeholder group to ensure wide dissemination of findings to effect change. Potential impacts will be upon service development, new models of care, training and workforce planning.

## Supplementary Material

Reviewer comments

Author's manuscript

## Data Availability

No data are available.
