# Supplementary material for: Mental Health Admissions to Paediatric Wards Study (MAPS): protocol of a prospective study of mental health admissions to paediatric wards in England using surveillance and qualitative methods
Source: BMJ Paediatr Open. 2024 Jan 25;8(1):e002186. doi: 10.1136/bmjpo-2023-002186 (PMC10824001; doi:10.1136/bmjpo-2023-002186)
Supplement: Author's manuscript [file bmjpo-2023-002186.draft_revisions.pdf]

BMJ Paediatrics Open

BMJ Paediatrics Open is committed to open peer review. As part of this commitment we make the peer review history of every article we publish publicly available.

When an article is published we post the peer reviewers' comments and the authors' responses online. We also post the versions of the paper that were used during peer review. These are the versions that the peer review comments apply to.

The versions of the paper that follow are the versions that were submitted during the peer review process. They are not the versions of record or the final published versions. They should not be cited or distributed as the published version of this manuscript.

BMJ Paediatrics Open is an open access journal and the full, final, typeset and author-corrected version of record of the manuscript is available on our site with no access controls, subscription charges or pay-per-view fees (http://bmjpaedsopen.bmj.com).

If you have any questions on BMJ Paediatrics Open's open peer review process please email info.bmjpo@bmj.com

BMJ Paediatrics Open

Mental Health Admissions to Paediatric Wards Study (MAPS): protocol of a prospective study of mental health admissions to paediatric wards in England using surveillance and qualitative methods.

Journal:	*BMJ Paediatrics Open*
Manuscript ID	bmjpo-2023-002186
Article Type:	Protocol
Date Submitted by the Author:	14-Jul-2023
Complete List of Authors:	Hudson, Lee; University College London Great Ormond Street Institute of Child Health<br>Vázquez-Vázquez, Adriana; University College London Great Ormond Street Institute of Child Health, Population, Policy and Practice<br>Gibson , Faith ; University College London Great Ormond Street Institute of Child Health; Great Ormond Street Hospital for Children NHS Trust, London, UK<br>Phillips , Kirsty ; University College London Great Ormond Street Institute of Child Health<br>Mathews , Gabrielle ; CYP Transformation Team, NHS England and NHS Improvement London, London, UK<br>Roberts , Helen ; University College London Great Ormond Street Institute of Child Health<br>Cornaglia , Francesca; Queen Mary University of London<br>Roland, Damian; University of Leicester, Health Sciences; University Hospitals of Leicester NHS Trust, Paediatric Emergency Medicine Leicester Academic (PEMLA) Group<br>Ward, Joseph ; University College London Great Ormond Street Institute of Child Health<br>Nicholls, Dasha; Imperial College London Department of Brain Sciences, Psychiatry<br>Elphinstone, Holly; University College London Great Ormond Street Institute of Child Health<br>Viner, Russell; University College London Great Ormond Street Institute of Child Health
Keywords:	Adolescent Health, Child Psychiatry, Data Collection, Epidemiology, Qualitative research

SCHOLARONE™
Manuscripts

I, the Submitting Author has the right to grant and does grant on behalf of all authors of the Work (as defined in the below author licence), an exclusive licence and/or a non-exclusive licence for contributions from authors who are: i) UK Crown employees; ii) where BMJ has agreed a CC-BY licence shall apply, and/or iii) in accordance with the terms applicable for US Federal Government officers or employees acting as part of their official duties; on a worldwide, perpetual, irrevocable, royalty-free basis to BMJ Publishing Group Ltd ("BMJ") its licensees and where the relevant Journal is co-owned by BMJ to the co-owners of the Journal, to publish the Work in this journal and any other BMJ products and to exploit all rights, as set out in our licence.

The Submitting Author accepts and understands that any supply made under these terms is made by BMJ to the Submitting Author unless you are acting as an employee on behalf of your employer or a postgraduate student of an affiliated institution which is paying any applicable article publishing charge ("APC") for Open Access articles. Where the Submitting Author wishes to make the Work available on an Open Access basis (and intends to pay the relevant APC), the terms of reuse of such Open Access shall be governed by a Creative Commons licence – details of these licences and which Creative Commons licence will apply to this Work are set out in our licence referred to above.

Other than as permitted in any relevant BMJ Author's Self Archiving Policies, I confirm this Work has not been accepted for publication elsewhere, is not being considered for publication elsewhere and does not duplicate material already published. I confirm all authors consent to publication of this Work and authorise the granting of this licence.

Confidential: For Review Only

Mental Health Admissions to Paediatric Wards Study (MAPS): protocol of a prospective study of mental health admissions to paediatric wards in England using surveillance and qualitative methods.

Lee Hudson[1,2*], Adriana Vázquez-Vázquez[1], Faith Gibson[1,2,3], Kirsty Phillips[1], Gabrielle Mathews[4], Helen Roberts[1], Francesca Cornaglia[5], Damian Roland[6], Joseph Ward[1], Dasha Nicholls[7], Holly Elphinstone[1], Russell Viner[1]

1. Population, Policy and Practice Research Programme, UCL Great Ormond Street Institute of Child Health, London, UK
2. Great Ormond Street Hospital for Children NHS Trust, London, UK
3. University of Surrey, Guildford, UK
4. CYP Transformation Team, NHS England and NHS Improvement London, London, UK
5. Queen Mary University of London, London, UK
6. University Hospitals of Leicester NHS Trust, Leicester, UK
7. Division of Psychiatry, Imperial College London, London, UK

*Corresponding author:
Lee Hudson
Population, Policy and Practice Research Programme, UCL Great Ormond Street Institute of Child Health, London, UK
l.hudson@ucl.ac.uk

Confidential: For Review Only

ABSTRACT

Introduction

Children and young people (CYP) presenting with a mental health (MH) crisis are frequently admitted to general acute paediatric wards as a place of safety. Prior to the pandemic, a survey in England showed that CYP occupied 6% of general paediatric inpatient beds due to a MH crisis, and there have been longstanding concerns about the quality of care to support these patients in this setting. We aim to generate a Theory of Change (ToC) model to improve the quality of care for CYP admitted to acute paediatric services after presenting in a MH crisis. Here we describe work packages (WP) 2 and 3 of the study, which have been granted ethics approval.

Methods and analysis

We will undertake a national (England), sequential, mixed methods study to inform a ToC framework alongside a stakeholder group consisting of patients, families/carers and health care professionals (HCPs). Our study consists of 4 work packages undertaken over 30 months. WP2 is limited to working with stakeholders to develop a data collection instrument and then use this in a prospective study of MH admissions over 6 months in 15 purposively recruited acute paediatric wards across England. WP3 consists of gathering the views of CYP, their families/carers and HCPs during admissions using semi-structured interviews.

Ethics and dissemination

WP2 and WP3 received ethical approval (Ref 23/LO/0349). We will publish the overall synthesis of data and the final ToC to improve care of CYP with MH crisis admitted to general acute paediatric settings. As co-producers of the ToC, we will work with our stakeholder group to ensure wide dissemination of findings. Potential impacts will be upon service development, new models of care, training and workforce planning.

What is already known on this topic

There is evidence that both the number of paediatric admissions and the severity of MH crisis in CYP have increased, and paediatric teams are experiencing challenges delivering safe care.

What this study hopes to add

- This study will characterise MH admissions in terms of sociodemographic factors, diagnoses and reasons admitted, as well as the factors influencing decisions to admit CYP to paediatric wards for primary MH problems.
- This study will describe the views and experiences of CYP, families/carers and HCPs during MH admissions to paediatric wards.
- This study will generate a ToC model to positively impact upon quality of care for CYP who are admitted to an acute paediatric service because of a MH crisis.

How this study might affect research, practice or policy
By producing a ToC approach, we expect to generate a system map to identify recommendations and transformation plans to share with policymakers, commissioners, service leads, and professionals.

1. INTRODUCTION

CYP presenting with a MH crisis are frequently admitted to general acute paediatric wards as a place of safety, despite not always having the resources or training (1, 2). Before the pandemic, a survey carried out in 2019 with 60% of acute paediatric services in England found that 6% of general paediatric beds were occupied by CYP with MH problems (3). Moreover, data from London suggest that the management of CYP with MH problems was one of the main challenges for acute children's services (3).

The rise in MH problems amongst CYP during the COVID-19 pandemic has been also well described (4,5). Recent national data report that being at high risk of MH problems rose from 1 in 9 in 2017 to 1 in 6 by 2021, with a doubling of the proportion of CYP at risk of eating problems over that same period (6). During the first wave, acute services became 'default providers' where community or inpatient CAMHS services were not accessible and during the third wave, admissions to acute wards appeared to peak (7). This mismatch of greater distress and reduced access led to increases in already unmet needs (8).

Although amplified by the pandemic, MH admissions to acute paediatric wards are a long-standing issue that has been identified as a leading safety and quality concern for acute paediatric providers for some years (1,3). The MAPS study aims to generate a Theory of Change (ToC) model to improve the quality of care for CYP admitted to acute paediatric services after presenting in a MH crisis. Our study consists of 4 work packages. Here we describe work package 2 (WP2) and work package 3 (WP3) (**Figure 1**), which have been granted ethics approval. WP1, limited to using national routine administrative data to identify and characterise trends in MH admissions in acute paediatric wards in England between 2015–2022, is currently undergoing ethics assessment, and the protocol will be described elsewhere when ethics processes are complete. For WP4 we will synthesise, with our stakeholder group, the data delivered by WP1-3 to create a ToC model for agreed impacts to inform service provision, potentially including the development of new pathways or models of care needed to improve the care of CYP admitted to acute wards. Overall, MAPS will look in detail at a serious healthcare problem for how CYP are treated and have access to and care for their MH. It will impact positively by providing the information needed to develop the way care is provided for CYP and families/carers.

INSERT FIGURE 1

2. METHODS AND ANALYSIS

We will use a ToC approach as our framework, which uses logic (quantitative) data and co-production (qualitative) data to map change. This approach has been applied to a range of areas of health and social care improvement settings (9-11).

Confidential: For Review Only

Study design

We will undertake a national, sequential, mixed methods study to inform a ToC framework alongside a stakeholder group consisting of patients, families/carers and HCPs. Three work packages will deliver the types of evidence needed to inform our ToC. Here we describe the study designs for WP2 and WP3.

WP2, consisting of WP2A and WP2B, aims to investigate factors influencing decisions to admit CYP to paediatric wards for primary MH problems, including why possible alternative services were not used, and to characterise the care given, treatment outcomes and subsequent service use. To achieve this aim, a detailed prospective data collection on MH admissions to acute paediatric wards in 15 centres in England will be carried out.

WP3 aims to explore the views and experiences of CYP admitted and the views and experiences of their families/carers and HCPs, concerning their admission, care and treatment. To fulfil this aim, we will 1) explore CYPs and their families'/carers' experience of their admission to a paediatric ward, including an understanding of the reasons for admission, the care and treatment received before and during this admission, 2) explore HCPs' experience of caring for CYP with primary MH problems, and their families/carers, to understand more fully their preparation for this role, as well as enablers and barriers to delivery of individualised patient-centred care and 3) determine recommendations for the improvement of care, treatment and outcomes of CYP and their families/carers.

WP2: Development of data collection instrument (WP2A) and data collection at paediatric wards (WP2B)

We used a modified Delphi approach, to work with our stakeholder group in the co-creation of our detailed prospective data collection tool (WP2A). Members of our stakeholder group and the Patient and Public Involvement and Engagement (PPIE) group were the experts in our Delphi.

First, the literature review informed an initial set of key domains which were modified after consultation with the group. Then, we developed the tool and sent it for comments/suggestions from the group. We incorporated the changes, and we built the online web tool form to enable data entry. We used the RedCap system in which data are directly imported into the secure UCL Data Safe Haven. For round two, we tested the instrument and online web form in three sites using a series of ‘dummy’ or fictitious patients. Feedback from this piloting was incorporated into the final instrument. **Table 1** shows the section headings and examples of questions that compose each section of the tool.

Table 1 MAPS tool

Section headings	Questions (examples)
Key facts about admission.	Date of admission and discharge.<br>Patient characteristics (age, sex, ethnicity).
Further detail about management during the admission.	Would you consider this admission as panned or unplanned?<br>Did the patient receive restraint during the admission?
Mental health assessment/support during admission.	Was the patient seen by a mental health professional on the ward?<br>Was guidance given to the in-patient team by a mental health professional during the admission?
Social aspects of the admission.	Was the patient known to a local authority social care team prior to admission?<br>Were social work or safeguarding issues raised during the admission?
Information about the lead-up/prior to admission.	Presentation to ED in the previous 3 months<br>Did the young person initially present alone?
Discharge.	Where was the patient discharged to?

https://mc.manuscriptcentral.com/bmjpo

	Did they get planned medical (paediatric follow-up) at discharge?
Overall perceptions of the admission (Likert scales with response options listed in ascending order [strongly disagree to strongly agree]).	The young person was involved in the decisions about their care during the admission.<br>The ward was prepared for this admission.

Using the tool, we will prospectively collect data on all primary MH admissions to 15 acute paediatric sites in England over 6 months (WP2B). We will purposively select these sites from children's hospitals and district general hospitals across different geographical regions, urban and rural, in England. A nominated paediatrician from each site will report data on all CYP admitted that meet the case definition over 6 months. Cases of interest will essentially be any CYP admitted to the acute paediatric ward for a primary MH crisis during the data collection period (**Table 2**).

Table 2 Inclusion criteria for MAPS WP2 and WP3

Inclusion criteria	Paediatric sites	CYP	Family/carers/friends	HCPs
WP2	We have already obtained outline agreements from approximately 30 sites that would welcome participation in this project. The purposively selected 15 sites will come from these 30 sites that have already signalled interest.<br><br>Site in which local NHS services opt-out processes are active.	Any CYP (≤ 18 years of age) admitted to the site for a primary MH crisis during the data collection period (6 months).		
WP3	Five acute paediatric sites will be selected from the 15 anonymised sites involved with WP2.	CYP aged 10 to 17 years (up to 18th birthday) who live in England.<br><br>CYP admitted to one of the five sites with a primary MH diagnosis.	Family members, carers/guardians or friends of CYP, who are 16 years and above, live in England, and have been identified by CYP as having played an important role in their care and admission*.	HCPs who are a member of the treating team for CYP working on one of the five selected paediatric wards.<br><br>HCPs self-identified by CYP as having played an important role in their care during their admission.

*Note that CYP can be included without identifying any family/carers for recruitment to the study.

In terms of sample size, our survey of 36 sites in Jan-March 2021 showed a median number of primary MH admissions per centre of 13 per month (7). We anticipate there will be a lower number post-pandemic of 8 patients per month across 15 sites, totalling 720 patients. We estimate that data will be collected on 90% of these, providing a sample of 650 for the study. We have not undertaken a formal power calculation but note that 650 patients provide a precision (95% CI) of +/- 2.3% for a proportion of 10% and +/- 3.5% for a proportion of 30% for the primary outcomes noted above (given school-age population approximately 8 million).

A summary of the WP2 procedure can be seen in **Figure 2**. Reporting of outcomes will be primarily descriptive, and we will minimise formal statistical testing.

INSERT FIGURE 2

WP3: Gathering the views of CYP, families/carers and HCPs

Understanding the experiences of CYP and their families/carers is fundamental to assessing the context of care. The NHS England-funded Amplified programme is providing evidence of the importance of and approaches to engaging with CYP, such as supporting and building participation to ensure CYP are involved in decision-making about their care and providing feedback on their experience (12). For CYP and families/carers experiencing vulnerabilities, we need to find ways of designing and promoting MH support that works for them. This requires understanding their experiences of care and service provision.

A multiple-case study will be our focus. Five acute paediatric sites will be selected from the 15 sites involved with WP2, with each site representing a case. We will apply a 'Diverse' case selection approach, which aims to ensure maximum variance of cases along relevant dimensions or criteria (13,14). Hence, the five sites selected will provide a rich and diverse sample of CYP experiencing MH problems, as well as family/carers and HCPs from varying disciplines.

Figure 3 illustrates the flow of steps which will be taken to identify and recruit CYP and **Table 1** the criteria for inclusion. A named staff lead for each of the five sites will support the identification of CYP to be approached. If consent to be contacted is given, a member of the research team would contact the CYP and parents/guardians of the CYP by phone to provide more information and gauge interest in the study. CYP will be asked to identify 1-2 family members/carers, as well as 1-2 HCPs from their paediatric centre to participate in data collection. The staff lead for each of the five sites will be asked to facilitate the recruitment of HCPs. For each case, we aim to recruit up to 7-8 CYP (total n=35-40), 8-9 family members/carers (total n=40-45) and 4-5 HCPs (total n=20-25). However, data collection will continue until a theory has emerged and the data set provides sufficient similarities and contrasts to the emerging theory (15).

INSERT FIGURE 3

Semi-structured interviews will be carried out and will be held virtually (MS Teams/Zoom) or by telephone. Interviews will allow for key questions to be explored with participants, and for a more in-depth exploration of experiences of care and service provision.

Our approach will be iterative-inductive analysis as there will be simultaneous sampling with collection and analysis of data, each informing the other. This will allow for structured and defensible flexibility in our study and maximise our ability to respond to theoretical sensitivity.

A summary of our study procedure for WP3 can be seen in **Figure 4**.

INSERT FIGURE 4

Confidential: For Review Only

ETHICS AND DISSEMINATION

WP2 and WP3 were approved by the London - West London & GTAC Research Ethics Committee (Ref 23/LO/0349). There are no direct risks to research participants. However, for WP2, we will not be asking for consent as the need to obtain consent would bias the sample and reduce generalisability. We have carefully considered the process of collecting data without consent. WP2 was presented to members of the Think4Brum, which is the youth advisory group for Forward Thinking Birmingham, and the GOSH Young Persons' Advisory Group for research as part of a PPIE initiative. Focus groups of 40 young people (aged <18) and parents was held to discuss the acceptability of the methods and the use of data without consent.

For WP3, participants will be carefully and fully informed of the study and asked to give written consent. Regarding dissemination, see WP4 of the study in **Figure 1**. As co-producers of the ToC, we will work with our stakeholder group to ensure wide dissemination of findings to effect change. Potential impacts will be upon service development, new models of care, training and workforce planning.

REFERENCES

1. Royal College of Paediatrics and Child Health. Role of paediatricians in supporting children and young people's mental health – position statement. Report, 2020. https://www.rcpch.ac.uk/resources/role-paediatricians-supporting-children-youngpeoples-mental-health-position-statement
2. Review of acute care services for children and young people: Healthy London Partnership, London, 2017. https://www.healthylondon.org/resource/peer-reviewacute-care-services-children-young-people/
3. Royal College of Paediatrics and Child Health. A snapshot of general paediatric services and workforce in the UK. Report, 2020.
4. Ford T, John A, Gunnell D. Mental health of children and young people during pandemic. BMJ. 2021 Mar 10;372:n614. doi: 10.1136/bmj.n614. Erratum in: BMJ. 2021 Mar 19;372:n765. PMID: 33692087.
5. Samji H, Wu J, Ladak A, Vossen C, Stewart E, Dove N, Long D, Snell G. Review: Mental health impacts of the COVID-19 pandemic on children and youth – a systematic review. Child Adolesc Ment Health. 2021 Aug 28. doi: 10.1111/camh.12501. Epub ahead of print. PMID: 34455683.
6. NHS Digitial. Mental Health of Children and Young People in England, 2021: Wave 2 follow up to the 2017 survey: NHS Digital, Health and Social Care Information Centre, 2021. https://files.digital.nhs.uk/97/B09EF8/mhcyp_2021_rep.pdf
7. Hudson LD, Chapman S, Street KN, Nicholls D, Roland D, Dubicka B, Gibson F, Mathews G, Viner RM. Increased admissions to paediatric wards with a primary mental health diagnosis: results of a survey of a network of eating disorder paediatricians in England. Arch Dis Child. 2021 Sep 14:archdischild-2021-322700. doi: 10.1136/archdischild-2021-322700. Epub ahead of print. PMID: 34521633.
8. Dubicka et al. (2017). Mental health services for children fail to meet soaring demand. BMJ 2017 Sep 29;358:j4254. Doi: 10.1136/bmj.j4254.
9. Wilkinson H, Hills D, Penn A, Barbrook-Johnson P (2021) Building a system-based Theory of Change using participatory systems mapping. Evaluation 27(1) 80-101.
10. Maini R, Mounier-Jack nS, Borghi J (2018) How to and how not to develop a theory of change to evaluate a complex intervention-reflections on an experience in the Democratic Republic of Congo BMJ Global Health 3: e000617.
11. Breuer E, Lee L, DeSilva M, Lund C (2016) Using theory of change to design and evaluate public health interventions-a systematic review Implementation Science 11:63.
12. Involving parents and carers in children and young people's mental health. YoungMinds 2020. https://www.youngminds.org.uk/media/h4uexcw1/ym-parent-carer-toolkit-2020.pdf

Confidential: For Review Only

13. Gerring, J., & Cojocaru, L. (2016). Selecting Cases for Intensive Analysis: A Diversity of Goals and Methods. Sociological Methods & Research, 45(3), 392–423. https://doi.org/10.1177/0049124116631692
14. Dey I. Grounding Grounded Theory: Guidelines for Qualitative Inquiry. San Diego: Academic Press; 1999.
15. Morse, M. and Field, A. (1995) Qualitative Research Methods for Health Professionals. Sage Publications, Thousand Oaks.

AUTHOR'S CONTRIBUTIONS

LH, FG, HR and RV were major contributors to the development of the study protocol. AVV drafted the manuscript. All authors contributed to revising the manuscript and approved the final version.

FUNDING STATEMENT

This study is supported by NIHR grant number 135036.

COMPETEING INTEREST STATEMENT

None declared.

Confidential: For Review Only

Figure 1. MAPS work package flow diagram

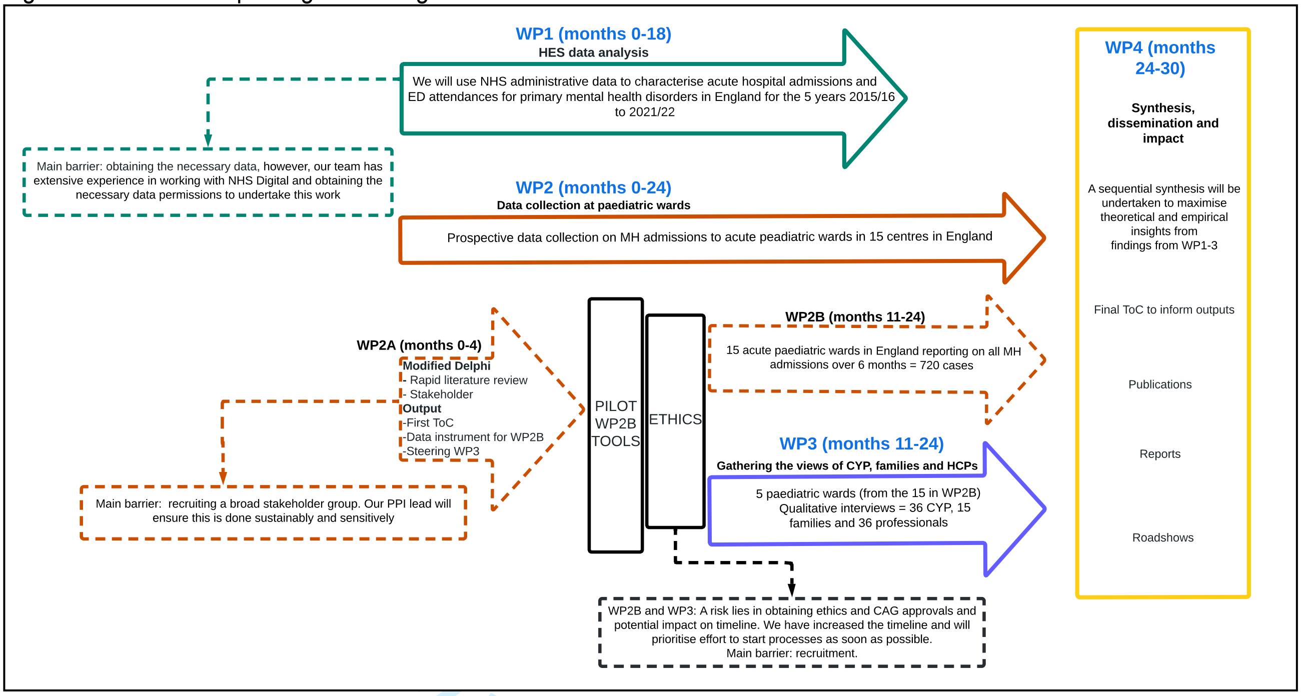

ED: Emergency department; MH: Mental Health; ToC: Theory of change; PPI: Patient and Public Involvement; CYP: Children and Young People; HCP: Health Care Professionals; CAG: Confidentially Advisory Group.

For peer review only

Figure 2. WP2 data flow diagram

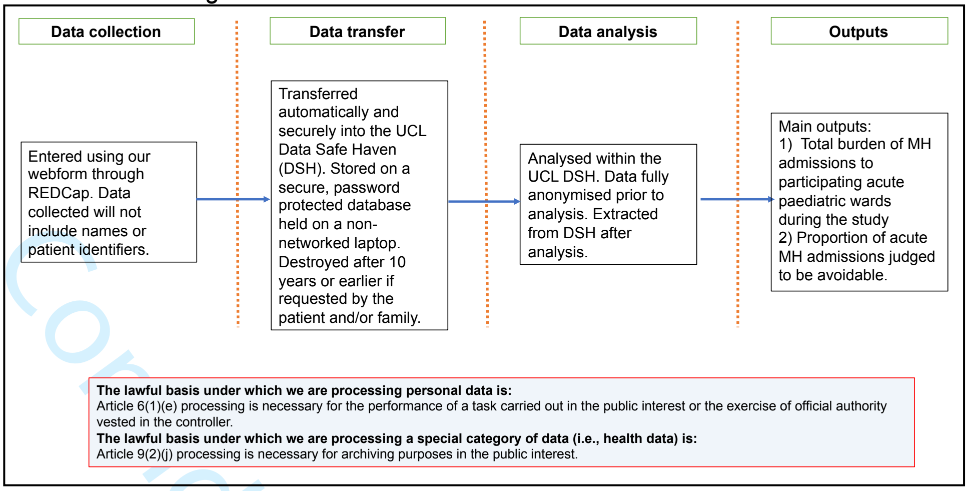

UCL: University College London; MH: Mental Health

Confidential: For Review Only

Figure 3. Recruitment flow chart for CYP

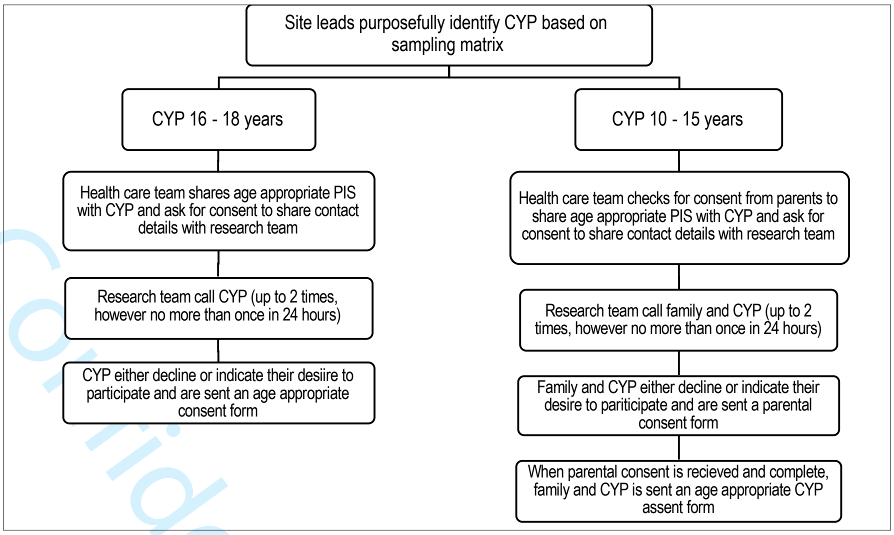

CYP: Children and Young People; PIS: Patient Information Sheet

Confidential: For Review Only

Figure 4. WP3 data flow diagram

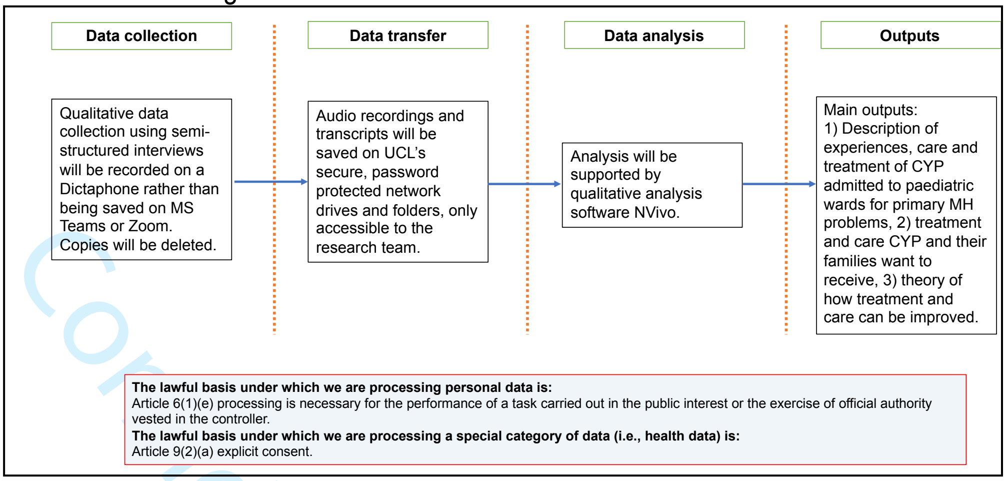

UCL: University College London; CYP: Children and Young People; MH: Mental Health

Confidential: For Review Only

BMJ Paediatrics Open

Mental Health Admissions to Paediatric Wards Study (MAPS): protocol of a prospective study of mental health admissions to paediatric wards in England using surveillance and qualitative methods.

Journal:	*BMJ Paediatrics Open*
Manuscript ID	bmjpo-2023-002186.R1
Article Type:	Protocol
Date Submitted by the Author:	28-Jul-2023

Complete List of Authors:	Hudson, Lee; University College London Great Ormond Street Institute of Child Health<br>Vázquez-Vázquez, Adriana; University College London Great Ormond Street Institute of Child Health, Population, Policy and Practice<br>Gibson , Faith ; University College London Great Ormond Street Institute of Child Health; Great Ormond Street Hospital for Children NHS Trust, London, UK<br>Phillips , Kirsty ; University College London Great Ormond Street Institute of Child Health<br>Mathews , Gabrielle ; CYP Transformation Team, NHS England and NHS Improvement London, London, UK<br>Roberts , Helen ; University College London Great Ormond Street Institute of Child Health<br>Cornaglia , Francesca; Queen Mary University of London<br>Roland, Damian; University of Leicester, Health Sciences; University Hospitals of Leicester NHS Trust, Paediatric Emergency Medicine Leicester Academic (PEMLA) Group<br>Ward, Joseph ; University College London Great Ormond Street Institute of Child Health<br>Nicholls, Dasha; Imperial College London Department of Brain Sciences, Psychiatry<br>Elphinstone, Holly; University College London Great Ormond Street Institute of Child Health<br>Viner, Russell; University College London Great Ormond Street Institute of Child Health

Keywords:	Adolescent Health, Child Psychiatry, Data Collection, Epidemiology, Qualitative research

SCHOLARONE™
Manuscripts

I, the Submitting Author has the right to grant and does grant on behalf of all authors of the Work (as defined in the below author licence), an exclusive licence and/or a non-exclusive licence for contributions from authors who are: i) UK Crown employees; ii) where BMJ has agreed a CC-BY licence shall apply, and/or iii) in accordance with the terms applicable for US Federal Government officers or employees acting as part of their official duties; on a worldwide, perpetual, irrevocable, royalty-free basis to BMJ Publishing Group Ltd ("BMJ") its licensees and where the relevant Journal is co-owned by BMJ to the co-owners of the Journal, to publish the Work in this journal and any other BMJ products and to exploit all rights, as set out in our licence.

The Submitting Author accepts and understands that any supply made under these terms is made by BMJ to the Submitting Author unless you are acting as an employee on behalf of your employer or a postgraduate student of an affiliated institution which is paying any applicable article publishing charge ("APC") for Open Access articles. Where the Submitting Author wishes to make the Work available on an Open Access basis (and intends to pay the relevant APC), the terms of reuse of such Open Access shall be governed by a Creative Commons licence – details of these licences and which Creative Commons licence will apply to this Work are set out in our licence referred to above.

Other than as permitted in any relevant BMJ Author's Self Archiving Policies, I confirm this Work has not been accepted for publication elsewhere, is not being considered for publication elsewhere and does not duplicate material already published. I confirm all authors consent to publication of this Work and authorise the granting of this licence.

Confidential: For Review Only

Mental Health Admissions to Paediatric Wards Study (MAPS): protocol of a prospective study of mental health admissions to paediatric wards in England using surveillance and qualitative methods.

Lee Hudson[1,2*], Adriana Vázquez-Vázquez[1], Faith Gibson[1,2,3], Kirsty Phillips[1], Gabrielle Mathews[4], Helen Roberts[1], Francesca Cornaglia[5], Damian Roland[6], Joseph Ward[1], Dasha Nicholls[7], Holly Elphinstone[1], Russell Viner[1]

1. Population, Policy and Practice Research Programme, UCL Great Ormond Street Institute of Child Health, London, UK
2. Great Ormond Street Hospital for Children NHS Trust, London, UK
3. University of Surrey, Guildford, UK
4. CYP Transformation Team, NHS England and NHS Improvement London, London, UK
5. Queen Mary University of London, London, UK
6. University Hospitals of Leicester NHS Trust, Leicester, UK
7. Division of Psychiatry, Imperial College London, London, UK

*Corresponding author:
Lee Hudson
Population, Policy and Practice Research Programme, UCL Great Ormond Street Institute of Child Health, London, UK
l.hudson@ucl.ac.uk

ABSTRACT

Introduction

Children and young people (CYP) presenting with a mental health (MH) crisis are frequently admitted to general acute paediatric wards as a place of safety. Prior to the pandemic, a survey in England showed that CYP occupied 6% of general paediatric inpatient beds due to a MH crisis, and there have been longstanding concerns about the quality of care to support these patients in this setting. We aim to generate a Theory of Change (ToC) model to improve the quality of care for CYP admitted to acute paediatric services after presenting in a MH crisis. Here we describe work packages (WP) 2 and 3 of the study, which have been granted ethics approval.

Methods and analysis

We will undertake a national (England), sequential, mixed methods study to inform a ToC framework alongside a stakeholder group consisting of patients, families/carers and health care professionals (HCPs). Our study consists of 4 work packages undertaken over 30 months. WP2 is limited to working with stakeholders to develop a data collection instrument and then use this in a prospective study of MH admissions over 6 months in 15 purposively recruited acute paediatric wards across England. WP3 consists of gathering the views of CYP, their families/carers and HCPs during admissions using semi-structured interviews.

Ethics and dissemination

WP2 and WP3 received ethical approval (Ref 23/LO/0349). We will publish the overall synthesis of data and the final ToC to improve care of CYP with MH crisis admitted to general acute paediatric settings. As co-producers of the ToC, we will work with our stakeholder group to ensure wide dissemination of findings. Potential impacts will be upon service development, new models of care, training and workforce planning.

What is already known on this topic

There is evidence that both the number of paediatric admissions and the severity of MH crisis in CYP have increased, and paediatric teams are experiencing challenges delivering safe care.

What this study hopes to add

Confidential: For Review Only

- This study will characterise MH admissions in terms of sociodemographic factors, diagnoses and reasons admitted, as well as the factors influencing decisions to admit CYP to paediatric wards for primary MH problems.
- This study will describe the views and experiences of CYP, families/carers and HCPs during MH admissions to paediatric wards.
- This study will generate a ToC model to positively impact upon quality of care for CYP who are admitted to an acute paediatric service because of a MH crisis.

How this study might affect research, practice or policy
By producing a ToC approach, we expect to generate a system map to identify recommendations and transformation plans to share with policymakers, commissioners, service leads, and professionals.

1. INTRODUCTION

CYP presenting with a MH crisis are frequently admitted to general acute paediatric wards as a place of safety, despite not always having the resources or training (1, 2). Before the pandemic, a survey carried out in 2019 with 60% of acute paediatric services in England found that 6% of general paediatric beds were occupied by CYP with MH problems (3). Moreover, data from London suggest that the management of CYP with MH problems was one of the main challenges for acute children's services (3).

The rise in MH problems amongst CYP during the COVID-19 pandemic has been also well described (4,5). Recent national data report that being at high risk of MH problems rose from 1 in 9 in 2017 to 1 in 6 by 2021, with a doubling of the proportion of CYP at risk of eating problems over that same period (6). During the first wave, acute services became 'default providers' where community or inpatient CAMHS services were not accessible and during the third wave, admissions to acute wards appeared to peak (7). This mismatch of greater distress and reduced access led to increases in already unmet needs (8).

Although amplified by the pandemic, MH admissions to acute paediatric wards are a long-standing issue that has been identified as a leading safety and quality concern for acute paediatric providers for some years (1,3). The MAPS study aims to generate a Theory of Change (ToC) model to improve the quality of care for CYP admitted to acute paediatric services after presenting in a MH crisis. Our study consists of 4 work packages. Here we describe work package 2 (WP2) and work package 3 (WP3) (**Figure 1**), which have been granted ethics approval. WP1, limited to using national routine administrative data to identify and characterise trends in MH admissions in acute paediatric wards in England between 2015–2022, is currently undergoing ethics assessment, and the protocol will be described elsewhere when ethics processes are complete. For WP4 we will synthesise, with our stakeholder group, the data delivered by WP1-3 to create a ToC model for agreed impacts to inform service provision, potentially including the development of new pathways or models of care needed to improve the care of CYP admitted to acute wards. Overall, MAPS will look in detail at a serious healthcare problem for how CYP are treated and have access to and care for their MH. It will impact positively by providing the information needed to develop the way care is provided for CYP and families/carers.

INSERT FIGURE 1

2. METHODS AND ANALYSIS

We will use a ToC approach as our framework, which uses logic (quantitative) data and co-production (qualitative) data to map change. This approach has been applied to a range of areas of health and social care improvement settings (9-11).

Confidential: For Review Only

Study design

We will undertake a national, sequential, mixed methods study to inform a ToC framework alongside a stakeholder group consisting of patients, families/carers and HCPs. Three work packages will deliver the types of evidence needed to inform our ToC. Here we describe the study designs for WP2 and WP3.

WP2, consisting of WP2A and WP2B, aims to investigate factors influencing decisions to admit CYP to paediatric wards for primary MH problems, including why possible alternative services were not used, and to characterise the care given, treatment outcomes and subsequent service use. To achieve this aim, a detailed prospective data collection on MH admissions to acute paediatric wards in 15 centres in England will be carried out.

WP3 aims to explore the views and experiences of CYP admitted and the views and experiences of their families/carers and HCPs, concerning their admission, care and treatment. To fulfil this aim, we will 1) explore CYPs and their families'/carers' experience of their admission to a paediatric ward, including an understanding of the reasons for admission, the care and treatment received before and during this admission, 2) explore HCPs' experience of caring for CYP with primary MH problems, and their families/carers, to understand more fully their preparation for this role, as well as enablers and barriers to delivery of individualised patient-centred care and 3) determine recommendations for the improvement of care, treatment and outcomes of CYP and their families/carers.

WP2: Development of data collection instrument (WP2A) and data collection at paediatric wards (WP2B)

We used a modified Delphi approach, to work with our stakeholder group in the co-creation of our detailed prospective data collection tool (WP2A). Members of our stakeholder group and the Patient and Public Involvement and Engagement (PPIE) group were the experts in our Delphi.

First, the literature review informed an initial set of key domains which were modified after consultation with the group. Then, we developed the tool and sent it for comments/suggestions from the group. We incorporated the changes, and we built the online web tool form to enable data entry. We used the RedCap system in which data are directly imported into the secure UCL Data Safe Haven. For round two, we tested the instrument and online web form in three sites using a series of 'dummy' or fictitious patients. Feedback from this piloting was incorporated into the final instrument. **Table 1** shows the section headings and examples of questions that compose each section of the tool.

Table 1 MAPS tool

Section headings	Questions (examples)
Key facts about admission.	Date of admission and discharge.<br>Patient characteristics (age, sex, ethnicity).
Further detail about management during the admission.	Would you consider this admission as panned or unplanned?<br>Did the patient receive restraint during the admission?
Mental health assessment/support during admission.	Was the patient seen by a mental health professional on the ward?<br>Was guidance given to the in-patient team by a mental health professional during the admission?
Social aspects of the admission.	Was the patient known to a local authority social care team prior to admission?<br>Were social work or safeguarding issues raised during the admission?
Information about the lead-up/prior to admission.	Presentation to ED in the previous 3 months<br>Did the young person initially present alone?
Discharge.	Where was the patient discharged to?

Confidential: For Review Only

	Did they get planned medical (paediatric follow-up) at discharge?
Overall perceptions of the admission (Likert scales with response options listed in ascending order [strongly disagree to strongly agree]).	The young person was involved in the decisions about their care during the admission.<br>The ward was prepared for this admission.

Using the tool, we will prospectively collect data on all primary MH admissions to 15 acute paediatric sites in England over 6 months (WP2B). We will purposively select these sites from children's hospitals and district general hospitals across different geographical regions, urban and rural, in England. A nominated paediatrician from each site will report data on all CYP admitted that meet the case definition over 6 months. Cases of interest will essentially be any CYP admitted to the acute paediatric ward for a primary MH crisis during the data collection period (**Table 2**).

Table 2 Inclusion criteria for MAPS WP2 and WP3

Inclusion criteria	Paediatric sites	CYP	Family/carers/friends	HCPs
WP2	We have already obtained outline agreements from approximately 30 sites that would welcome participation in this project. The purposively selected 15 sites will come from these 30 sites that have already signalled interest.<br><br>Site in which local NHS services opt-out processes are active.	Any CYP (≤ 18 years of age) admitted to the site for a primary MH crisis during the data collection period (6 months).		
WP3	Five acute paediatric sites will be selected from the 15 anonymised sites involved with WP2.	CYP aged 10 to 17 years (up to 18th birthday) who live in England.<br><br>CYP admitted to one of the five sites with a primary MH diagnosis.	Family members, carers/guardians or friends of CYP, who are 16 years and above, live in England, and have been identified by CYP as having played an important role in their care and admission*.	HCPs who are a member of the treating team for CYP working on one of the five selected paediatric wards.<br><br>HCPs self-identified by CYP as having played an important role in their care during their admission.

*Note that CYP can be included without identifying any family/carers for recruitment to the study.

In terms of sample size, our survey of 36 sites in Jan-March 2021 showed a median number of primary MH admissions per centre of 13 per month (7). We anticipate there will be a lower number post-pandemic of 8 patients per month across 15 sites, totalling 720 patients. We estimate that data will be collected on 90% of these, providing a sample of 650 for the study. We have not undertaken a formal power calculation but note that 650 patients provide a precision (95% CI) of +/- 2.3% for a proportion of 10% and +/- 3.5% for a proportion of 30% for the primary outcomes noted above (given school-age population approximately 8 million).

Confidential: For Review Only

A summary of the WP2 procedure can be seen in **Figure 2**. Reporting of outcomes will be primarily descriptive, and we will minimise formal statistical testing.

INSERT FIGURE 2

WP3: Gathering the views of CYP, families/carers and HCPs
Understanding the experiences of CYP and their families/carers is fundamental to assessing the context of care. The NHS England-funded Amplified programme is providing evidence of the importance of and approaches to engaging with CYP, such as supporting and building participation to ensure CYP are involved in decision-making about their care and providing feedback on their experience (12). For CYP and families/carers experiencing vulnerabilities, we need to find ways of designing and promoting MH support that works for them. This requires understanding their experiences of care and service provision.

A multiple-case study will be our focus. Five acute paediatric sites will be selected from the 15 sites involved with WP2, with each site representing a case. We will apply a 'Diverse' case selection approach, which aims to ensure maximum variance of cases along relevant dimensions or criteria (13,14). Hence, the five sites selected will provide a rich and diverse sample of CYP experiencing MH problems, as well as family/carers and HCPs from varying disciplines.

Figure 3 illustrates the flow of steps which will be taken to identify and recruit CYP and **Table 1** the criteria for inclusion. A named staff lead for each of the five sites will support the identification of CYP to be approached. If consent to be contacted is given, a member of the research team would contact the CYP and parents/guardians of the CYP by phone to provide more information and gauge interest in the study. CYP will be asked to identify 1-2 family members/carers, as well as 1-2 HCPs from their paediatric centre to participate in data collection. The staff lead for each of the five sites will be asked to facilitate the recruitment of HCPs. For each case, we aim to recruit up to 7-8 CYP (total n=35-40), 8-9 family members/carers (total n=40-45) and 4-5 HCPs (total n=20-25). However, data collection will continue until a theory has emerged and the data set provides sufficient similarities and contrasts to the emerging theory (15).

INSERT FIGURE 3

Semi-structured interviews will be carried out and will be held virtually (MS Teams/Zoom) or by telephone. Interviews will allow for key questions to be explored with participants, and for a more in-depth exploration of experiences of care and service provision.

Our approach will be iterative-inductive analysis as there will be simultaneous sampling with collection and analysis of data, each informing the other. This will allow for structured and defensible flexibility in our study and maximise our ability to respond to theoretical sensitivity.

A summary of our study procedure for WP3 can be seen in **Figure 4**.

INSERT FIGURE 4

Confidential: For Review Only

ETHICS AND DISSEMINATION

WP2 and WP3 were approved by the London - West London & GTAC Research Ethics Committee (Ref 23/LO/0349). There are no direct risks to research participants. However, for WP2, we will not be asking for consent as the need to obtain consent would bias the sample and reduce generalisability. We have carefully considered the process of collecting data without consent. WP2 was presented to members of the Think4Brum, which is the youth advisory group for Forward Thinking Birmingham, and the GOSH Young Persons' Advisory Group for research as part of a PPIE initiative. Focus groups of 40 young people (aged <18) and parents was held to discuss the acceptability of the methods and the use of data without consent.

For WP3, participants will be carefully and fully informed of the study and asked to give written consent. Regarding dissemination, see WP4 of the study in **Figure 1**. As co-producers of the ToC, we will work with our stakeholder group to ensure wide dissemination of findings to effect change. Potential impacts will be upon service development, new models of care, training and workforce planning.

REFERENCES

1. Royal College of Paediatrics and Child Health. Role of paediatricians in supporting children and young people's mental health – position statement. Report, 2020. https://www.rcpch.ac.uk/resources/role-paediatricians-supporting-children-youngpeoples-mental-health-position-statement
2. Review of acute care services for children and young people: Healthy London Partnership, London, 2017. https://www.healthylondon.org/resource/peer-reviewacute-care-services-children-young-people/
3. Royal College of Paediatrics and Child Health. A snapshot of general paediatric services and workforce in the UK. Report, 2020.
4. Ford T, John A, Gunnell D. Mental health of children and young people during pandemic. BMJ. 2021 Mar 10;372:n614. doi: 10.1136/bmj.n614. Erratum in: BMJ. 2021 Mar 19;372:n765. PMID: 33692087.
5. Samji H, Wu J, Ladak A, Vossen C, Stewart E, Dove N, Long D, Snell G. Review: Mental health impacts of the COVID-19 pandemic on children and youth – a systematic review. Child Adolesc Ment Health. 2021 Aug 28. doi: 10.1111/camh.12501. Epub ahead of print. PMID: 34455683.
6. NHS Digitial. Mental Health of Children and Young People in England, 2021: Wave 2 follow up to the 2017 survey: NHS Digital, Health and Social Care Information Centre, 2021. https://files.digital.nhs.uk/97/B09EF8/mhcyp_2021_rep.pdf
7. Hudson LD, Chapman S, Street KN, Nicholls D, Roland D, Dubicka B, Gibson F, Mathews G, Viner RM. Increased admissions to paediatric wards with a primary mental health diagnosis: results of a survey of a network of eating disorder paediatricians in England. Arch Dis Child. 2021 Sep 14:archdischild-2021-322700. doi: 10.1136/archdischild-2021-322700. Epub ahead of print. PMID: 34521633.
8. Dubicka et al. (2017). Mental health services for children fail to meet soaring demand. BMJ 2017 Sep 29;358:j4254. Doi: 10.1136/bmj.j4254.
9. Wilkinson H, Hills D, Penn A, Barbrook-Johnson P (2021) Building a system-based Theory of Change using participatory systems mapping. Evaluation 27(1) 80-101.
10. Maini R, Mounier-Jack nS, Borghi J (2018) How to and how not to develop a theory of change to evaluate a complex intervention-reflections on an experience in the Democratic Republic of Congo BMJ Global Health 3: e000617.
11. Breuer E, Lee L, DeSilva M, Lund C (2016) Using theory of change to design and evaluate public health interventions-a systematic review Implementation Science 11:63.
12. Involving parents and carers in children and young people's mental health. YoungMinds 2020. https://www.youngminds.org.uk/media/h4uexcw1/ym-parent-carer-toolkit-2020.pdf

Confidential: For Review Only

13. Gerring, J., & Cojocaru, L. (2016). Selecting Cases for Intensive Analysis: A Diversity of Goals and Methods. Sociological Methods & Research, 45(3), 392–423. https://doi.org/10.1177/0049124116631692
14. Dey I. Grounding Grounded Theory: Guidelines for Qualitative Inquiry. San Diego: Academic Press; 1999.
15. Morse, M. and Field, A. (1995) Qualitative Research Methods for Health Professionals. Sage Publications, Thousand Oaks.

AUTHOR'S CONTRIBUTIONS

LH, FG, HR and RV were major contributors to the development of the study protocol. AVV drafted the manuscript. All authors contributed to revising the manuscript and approved the final version.

FUNDING STATEMENT

This study is supported by NIHR grant number 135036.

COMPETEING INTEREST STATEMENT

None declared.

Confidential: For Review Only

Figure 1. MAPS work package flow diagram

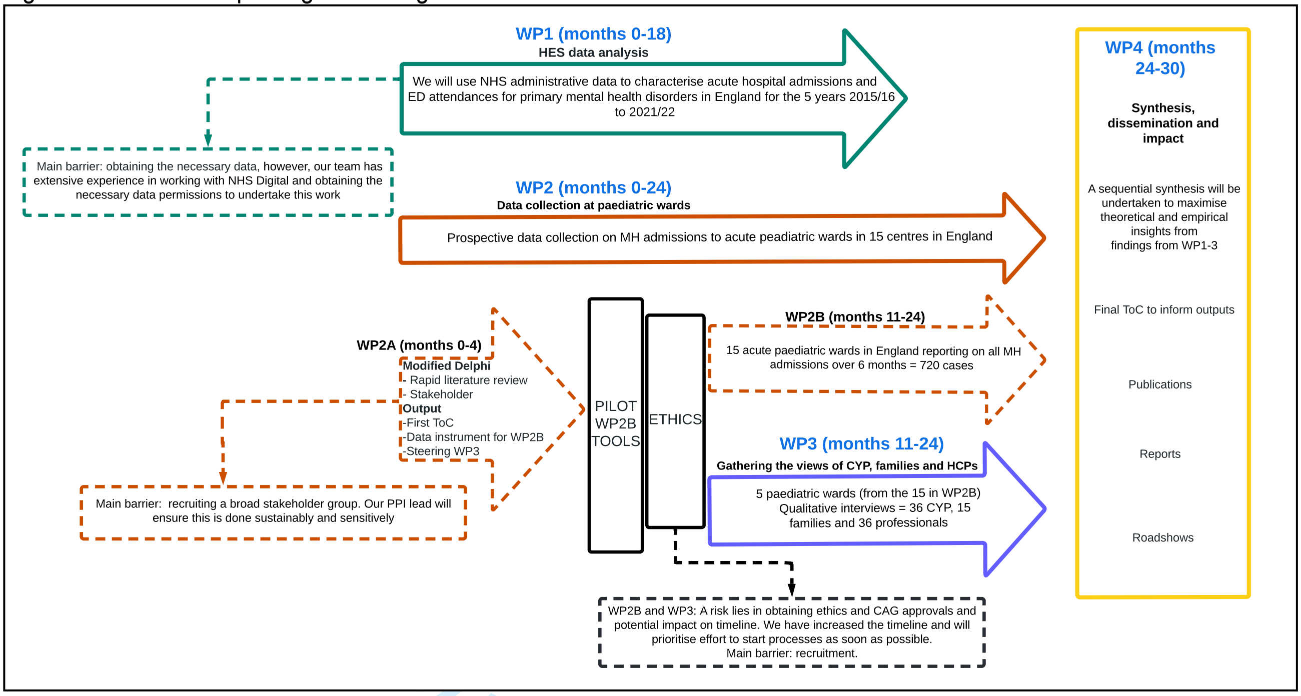

ED: Emergency department; MH: Mental Health; ToC: Theory of change; PPI: Patient and Public Involvement; CYP: Children and Young People; HCP: Health Care Professionals; CAG: Confidentially Advisory Group.

For Peer Review Only

Figure 2. WP2 data flow diagram

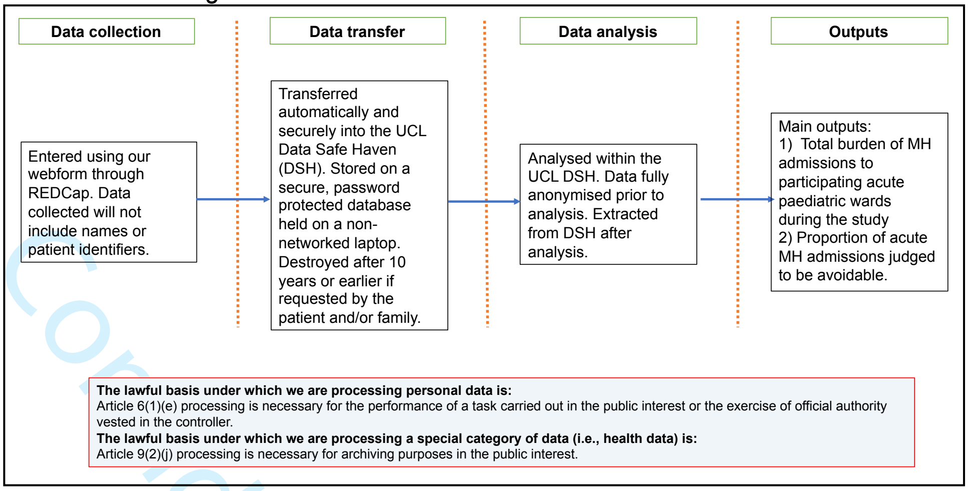

UCL: University College London; MH: Mental Health

Confidential: For Review Only

Figure 3. Recruitment flow chart for CYP

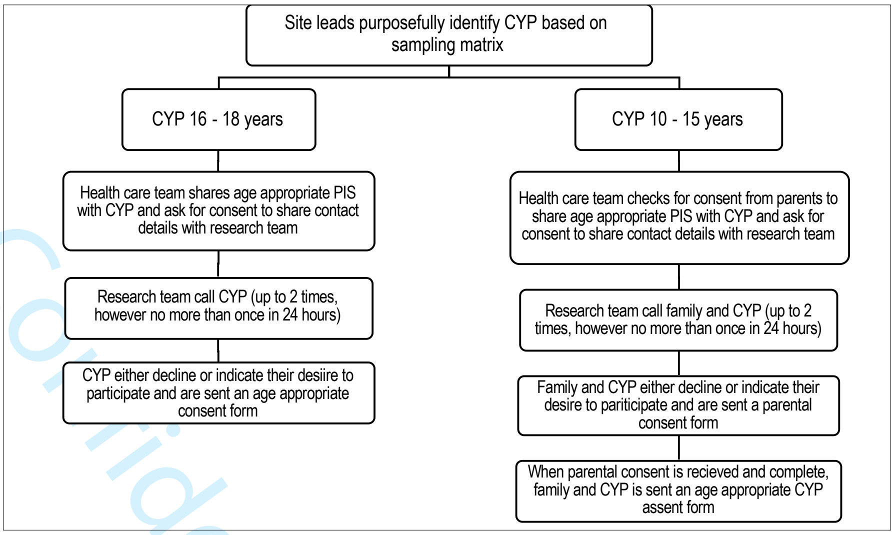

CYP: Children and Young People; PIS: Patient Information Sheet

Confidential: For Review Only

Figure 4. WP3 data flow diagram

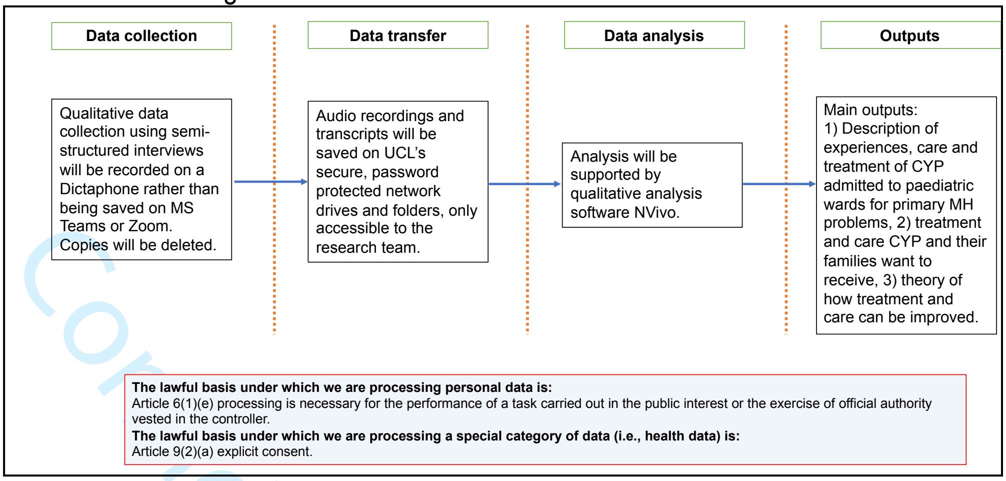

UCL: University College London; CYP: Children and Young People; MH: Mental Health

Confidential: For Review Only

BMJ Paediatrics Open

Mental Health Admissions to Paediatric Wards Study (MAPS): protocol of a prospective study of mental health admissions to paediatric wards in England using surveillance and qualitative methods.

Journal:	*BMJ Paediatrics Open*
Manuscript ID	bmjpo-2023-002186.R2
Article Type:	Protocol
Date Submitted by the Author:	08-Aug-2023
Complete List of Authors:	Hudson, Lee; University College London Great Ormond Street Institute of Child Health<br>Vázquez-Vázquez, Adriana; University College London Great Ormond Street Institute of Child Health, Population, Policy and Practice<br>Gibson , Faith ; University College London Great Ormond Street Institute of Child Health; Great Ormond Street Hospital for Children NHS Trust, London, UK<br>Phillips , Kirsty ; University College London Great Ormond Street Institute of Child Health<br>Mathews , Gabrielle ; CYP Transformation Team, NHS England and NHS Improvement London, London, UK<br>Roberts , Helen ; University College London Great Ormond Street Institute of Child Health<br>Cornaglia , Francesca; Queen Mary University of London<br>Roland, Damian; University of Leicester, Health Sciences; University Hospitals of Leicester NHS Trust, Paediatric Emergency Medicine Leicester Academic (PEMLA) Group<br>Ward, Joseph ; University College London Great Ormond Street Institute of Child Health<br>Nicholls, Dasha; Imperial College London Department of Brain Sciences, Psychiatry<br>Elphinstone, Holly; University College London Great Ormond Street Institute of Child Health<br>Viner, Russell; University College London Great Ormond Street Institute of Child Health
Keywords:	Adolescent Health, Child Psychiatry, Data Collection, Epidemiology, Qualitative research

SCHOLARONE™
Manuscripts

I, the Submitting Author has the right to grant and does grant on behalf of all authors of the Work (as defined in the below author licence), an exclusive licence and/or a non-exclusive licence for contributions from authors who are: i) UK Crown employees; ii) where BMJ has agreed a CC-BY licence shall apply, and/or iii) in accordance with the terms applicable for US Federal Government officers or employees acting as part of their official duties; on a worldwide, perpetual, irrevocable, royalty-free basis to BMJ Publishing Group Ltd ("BMJ") its licensees and where the relevant Journal is co-owned by BMJ to the co-owners of the Journal, to publish the Work in this journal and any other BMJ products and to exploit all rights, as set out in our licence.

The Submitting Author accepts and understands that any supply made under these terms is made by BMJ to the Submitting Author unless you are acting as an employee on behalf of your employer or a postgraduate student of an affiliated institution which is paying any applicable article publishing charge ("APC") for Open Access articles. Where the Submitting Author wishes to make the Work available on an Open Access basis (and intends to pay the relevant APC), the terms of reuse of such Open Access shall be governed by a Creative Commons licence – details of these licences and which Creative Commons licence will apply to this Work are set out in our licence referred to above.

Other than as permitted in any relevant BMJ Author's Self Archiving Policies, I confirm this Work has not been accepted for publication elsewhere, is not being considered for publication elsewhere and does not duplicate material already published. I confirm all authors consent to publication of this Work and authorise the granting of this licence.

Confidential: For Review Only

Mental Health Admissions to Paediatric Wards Study (MAPS): protocol of a prospective study of mental health admissions to paediatric wards in England using surveillance and qualitative methods.

Lee Hudson[1,2*], Adriana Vázquez-Vázquez[1], Faith Gibson[1,2,3], Kirsty Phillips[1], Gabrielle Mathews[4], Helen Roberts[1], Francesca Cornaglia[5], Damian Roland[6], Joseph Ward[1], Dasha Nicholls[7], Holly Elphinstone[1], Russell Viner[1]

1. Population, Policy and Practice Research Programme, UCL Great Ormond Street Institute of Child Health, London, UK
2. Great Ormond Street Hospital for Children NHS Trust, London, UK
3. University of Surrey, Guildford, UK
4. CYP Transformation Team, NHS England and NHS Improvement London, London, UK
5. Queen Mary University of London, London, UK
6. University Hospitals of Leicester NHS Trust, Leicester, UK
7. Division of Psychiatry, Imperial College London, London, UK

*Corresponding author:
Lee Hudson
Population, Policy and Practice Research Programme, UCL Great Ormond Street Institute of Child Health, London, UK
l.hudson@ucl.ac.uk

Confidential: For Review Only

ABSTRACT

Introduction

Children and young people (CYP) presenting with a mental health (MH) crisis are frequently admitted to general acute paediatric wards as a place of safety. Prior to the pandemic, a survey in England showed that CYP occupied 6% of general paediatric inpatient beds due to a MH crisis, and there have been longstanding concerns about the quality of care to support these patients in this setting. We aim to generate a Theory of Change (ToC) model to improve the quality of care for CYP admitted to acute paediatric services after presenting in a MH crisis. Here we describe work packages (WP) 2 and 3 of the study, which have been granted ethics approval.

Methods and analysis

We will undertake a national (England), sequential, mixed methods study to inform a ToC framework alongside a stakeholder group consisting of patients, families/carers and health care professionals (HCPs). Our study consists of 4 work packages undertaken over 30 months. WP2 is limited to working with stakeholders to develop a data collection instrument and then use this in a prospective study of MH admissions over 6 months in 15 purposively recruited acute paediatric wards across England. WP3 consists of gathering the views of CYP, their families/carers and HCPs during admissions using semi-structured interviews.

Ethics and dissemination

WP2 and WP3 received ethical approval (Ref 23/LO/0349). We will publish the overall synthesis of data and the final ToC to improve care of CYP with MH crisis admitted to general acute paediatric settings. As co-producers of the ToC, we will work with our stakeholder group to ensure wide dissemination of findings. Potential impacts will be upon service development, new models of care, training and workforce planning.

What is already known on this topic

There is evidence that both the number of paediatric admissions and the severity of MH crisis in CYP have increased, and paediatric teams are experiencing challenges delivering safe care.

What this study hopes to add

- This study will characterise MH admissions in terms of sociodemographic factors, diagnoses and reasons admitted, as well as the factors influencing decisions to admit CYP to paediatric wards for primary MH problems.
- This study will describe the views and experiences of CYP, families/carers and HCPs during MH admissions to paediatric wards.
- This study will generate a ToC model to positively impact upon quality of care for CYP who are admitted to an acute paediatric service because of a MH crisis.

How this study might affect research, practice or policy
By producing a ToC approach, we expect to generate a system map to identify recommendations and transformation plans to share with policymakers, commissioners, service leads, and professionals.

1. INTRODUCTION
CYP presenting with a MH crisis are frequently admitted to general acute paediatric wards as a place of safety, despite not always having the resources or training (1, 2). Before the pandemic, a survey carried out in 2019 with 60% of acute paediatric services in England found that 6% of general paediatric beds were occupied by CYP with MH problems (3). Moreover, data from London suggest that the management of CYP with MH problems was one of the main challenges for acute children's services (3).

The rise in MH problems amongst CYP during the COVID-19 pandemic has been also well described (4,5). Recent national data report that being at high risk of MH problems rose from 1 in 9 in 2017 to 1 in 6 by 2021, with a doubling of the proportion of CYP at risk of eating problems over that same period (6). During the first wave, acute services became 'default providers' where community or inpatient CAMHS services were not accessible and during the third wave, admissions to acute wards appeared to peak (7). This mismatch of greater distress and reduced access led to increases in already unmet needs (8).

Although amplified by the pandemic, MH admissions to acute paediatric wards are a long-standing issue that has been identified as a leading safety and quality concern for acute paediatric providers for some years (1,3). The MAPS study aims to generate a Theory of Change (ToC) model to improve the quality of care for CYP admitted to acute paediatric services after presenting in a MH crisis. Our study consists of 4 work packages. Here we describe work package 2 (WP2) and work package 3 (WP3) (**Figure 1**), which have been granted ethics approval. WP1, limited to using national routine administrative data to identify and characterise trends in MH admissions in acute paediatric wards in England between 2015–2022, is currently undergoing ethics assessment, and the protocol will be described elsewhere when ethics processes are complete. For WP4 we will synthesise, with our stakeholder group, the data delivered by WP1-3 to create a ToC model for agreed impacts to inform service provision, potentially including the development of new pathways or models of care needed to improve the care of CYP admitted to acute wards. Overall, MAPS will look in detail at a serious healthcare problem for how CYP are treated and have access to and care for their MH. It will impact positively by providing the information needed to develop the way care is provided for CYP and families/carers.

INSERT FIGURE 1

2. METHODS AND ANALYSIS
We will use a ToC approach as our framework, which uses logic (quantitative) data and co-production (qualitative) data to map change. This approach has been applied to a range of areas of health and social care improvement settings (9-11).

Confidential: For Review Only

Confidential: For Review Only

Study design

We will undertake a national, sequential, mixed methods study to inform a ToC framework alongside a stakeholder group consisting of patients, families/carers and HCPs. Three work packages will deliver the types of evidence needed to inform our ToC. Here we describe the study designs for WP2 and WP3.

WP2, consisting of WP2A and WP2B, aims to investigate factors influencing decisions to admit CYP to paediatric wards for primary MH problems, including why possible alternative services were not used, and to characterise the care given, treatment outcomes and subsequent service use. To achieve this aim, a detailed prospective data collection on MH admissions to acute paediatric wards in 15 centres in England will be carried out.

WP3 aims to explore the views and experiences of CYP admitted and the views and experiences of their families/carers and HCPs, concerning their admission, care and treatment. To fulfil this aim, we will 1) explore CYPs and their families'/carers' experience of their admission to a paediatric ward, including an understanding of the reasons for admission, the care and treatment received before and during this admission, 2) explore HCPs' experience of caring for CYP with primary MH problems, and their families/carers, to understand more fully their preparation for this role, as well as enablers and barriers to delivery of individualised patient-centred care and 3) determine recommendations for the improvement of care, treatment and outcomes of CYP and their families/carers.

WP2: Development of data collection instrument (WP2A) and data collection at paediatric wards (WP2B)

We used a modified Delphi approach, to work with our stakeholder group in the co-creation of our detailed prospective data collection tool (WP2A). Members of our stakeholder group and the Patient and Public Involvement and Engagement (PPIE) group were the experts in our Delphi.

First, the literature review informed an initial set of key domains which were modified after consultation with the group. Then, we developed the tool and sent it for comments/suggestions from the group. We incorporated the changes, and we built the online web tool form to enable data entry. We used the RedCap system in which data are directly imported into the secure UCL Data Safe Haven. For round two, we tested the instrument and online web form in three sites using a series of 'dummy' or fictitious patients. Feedback from this piloting was incorporated into the final instrument. **Table 1** shows the section headings and examples of questions that compose each section of the tool.

Table 1 MAPS tool

Section headings	Questions (examples)
Key facts about admission.	Date of admission and discharge.<br>Patient characteristics (age, sex, ethnicity).
Further detail about management during the admission.	Would you consider this admission as panned or unplanned?<br>Did the patient receive restraint during the admission?
Mental health assessment/support during admission.	Was the patient seen by a mental health professional on the ward?<br>Was guidance given to the in-patient team by a mental health professional during the admission?
Social aspects of the admission.	Was the patient known to a local authority social care team prior to admission?<br>Were social work or safeguarding issues raised during the admission?
Information about the lead-up/prior to admission.	Presentation to ED in the previous 3 months<br>Did the young person initially present alone?
Discharge.	Where was the patient discharged to?

	Did they get planned medical (paediatric follow-up) at discharge?
Overall perceptions of the admission (Likert scales with response options listed in ascending order [strongly disagree to strongly agree]).	The young person was involved in the decisions about their care during the admission.<br>The ward was prepared for this admission.

Using the tool, we will prospectively collect data on all primary MH admissions to 15 acute paediatric sites in England over 6 months (WP2B). We will purposively select these sites from children's hospitals and district general hospitals across different geographical regions, urban and rural, in England. A nominated paediatrician from each site will report data on all CYP admitted that meet the case definition over 6 months. Cases of interest will essentially be any CYP admitted to the acute paediatric ward for a primary MH crisis during the data collection period (**Table 2**).

Table 2 Inclusion criteria for MAPS WP2 and WP3

Inclusion criteria	Paediatric sites	CYP	Family/carers/friends	HCPs
WP2	We have already obtained outline agreements from approximately 30 sites that would welcome participation in this project. The purposively selected 15 sites will come from these 30 sites that have already signalled interest.<br><br>Site in which local NHS services opt-out processes are active.	Any CYP (≤ 18 years of age) admitted to the site for a primary MH crisis during the data collection period (6 months).		
WP3	Five acute paediatric sites will be selected from the 15 anonymised sites involved with WP2.	CYP aged 10 to 17 years (up to 18th birthday) who live in England.<br><br>CYP admitted to one of the five sites with a primary MH diagnosis.	Family members, carers/guardians or friends of CYP, who are 16 years and above, live in England, and have been identified by CYP as having played an important role in their care and admission*.	HCPs who are a member of the treating team for CYP working on one of the five selected paediatric wards.<br><br>HCPs self-identified by CYP as having played an important role in their care during their admission.

*Note that CYP can be included without identifying any family/carers for recruitment to the study.

In terms of sample size, our survey of 36 sites in Jan-March 2021 showed a median number of primary MH admissions per centre of 13 per month (7). We anticipate there will be a lower number post-pandemic of 8 patients per month across 15 sites, totalling 720 patients. We estimate that data will be collected on 90% of these, providing a sample of 650 for the study. We have not undertaken a formal power calculation but note that 650 patients provide a precision (95% CI) of +/- 2.3% for a proportion of 10% and +/- 3.5% for a proportion of 30% for the primary outcomes noted above (given school-age population approximately 8 million).

Confidential: For Review Only

A summary of the WP2 procedure can be seen in **Figure 2**. Reporting of outcomes will be primarily descriptive, and we will minimise formal statistical testing.

INSERT FIGURE 2

WP3: Gathering the views of CYP, families/carers and HCPs

Understanding the experiences of CYP and their families/carers is fundamental to assessing the context of care. The NHS England-funded Amplified programme is providing evidence of the importance of and approaches to engaging with CYP, such as supporting and building participation to ensure CYP are involved in decision-making about their care and providing feedback on their experience (12). For CYP and families/carers experiencing vulnerabilities, we need to find ways of designing and promoting MH support that works for them. This requires understanding their experiences of care and service provision.

A multiple-case study will be our focus. Five acute paediatric sites will be selected from the 15 sites involved with WP2, with each site representing a case. We will apply a 'Diverse' case selection approach, which aims to ensure maximum variance of cases along relevant dimensions or criteria (13,14). Hence, the five sites selected will provide a rich and diverse sample of CYP experiencing MH problems, as well as family/carers and HCPs from varying disciplines.

Figure 3 illustrates the flow of steps which will be taken to identify and recruit CYP and **Table 1** the criteria for inclusion. A named staff lead for each of the five sites will support the identification of CYP to be approached. If consent to be contacted is given, a member of the research team would contact the CYP and parents/guardians of the CYP by phone to provide more information and gauge interest in the study. CYP will be asked to identify 1-2 family members/carers, as well as 1-2 HCPs from their paediatric centre to participate in data collection. The staff lead for each of the five sites will be asked to facilitate the recruitment of HCPs. For each case, we aim to recruit up to 7-8 CYP (total n=35-40), 8-9 family members/carers (total n=40-45) and 4-5 HCPs (total n=20-25). However, data collection will continue until a theory has emerged and the data set provides sufficient similarities and contrasts to the emerging theory (15).

INSERT FIGURE 3

Semi-structured interviews will be carried out and will be held virtually (MS Teams/Zoom) or by telephone. Interviews will allow for key questions to be explored with participants, and for a more in-depth exploration of experiences of care and service provision.

Our approach will be iterative-inductive analysis as there will be simultaneous sampling with collection and analysis of data, each informing the other. This will allow for structured and defensible flexibility in our study and maximise our ability to respond to theoretical sensitivity.

A summary of our study procedure for WP3 can be seen in **Figure 4**.

INSERT FIGURE 4

Confidential: For Review Only

ETHICS AND DISSEMINATION

WP2 and WP3 were approved by the London - West London & GTAC Research Ethics Committee (Ref 23/LO/0349). There are no direct risks to research participants. However, for WP2, we will not be asking for consent as the need to obtain consent would bias the sample and reduce generalisability. We have carefully considered the process of collecting data without consent. WP2 was presented to members of the Think4Brum, which is the youth advisory group for Forward Thinking Birmingham, and the GOSH Young Persons' Advisory Group for research as part of a PPIE initiative. Focus groups of 40 young people (aged <18) and parents was held to discuss the acceptability of the methods and the use of data without consent.

For WP3, participants will be carefully and fully informed of the study and asked to give written consent. Regarding dissemination, see WP4 of the study in **Figure 1**. As co-producers of the ToC, we will work with our stakeholder group to ensure wide dissemination of findings to effect change. Potential impacts will be upon service development, new models of care, training and workforce planning.

REFERENCES

1. Royal College of Paediatrics and Child Health. Role of paediatricians in supporting children and young people's mental health – position statement. Report, 2020. https://www.rcpch.ac.uk/resources/role-paediatricians-supporting-children-youngpeoples-mental-health-position-statement
2. Review of acute care services for children and young people: Healthy London Partnership, London, 2017. https://www.healthylondon.org/resource/peer-reviewacute-care-services-children-young-people/
3. Royal College of Paediatrics and Child Health. A snapshot of general paediatric services and workforce in the UK. Report, 2020.
4. Ford T, John A, Gunnell D. Mental health of children and young people during pandemic. BMJ. 2021 Mar 10;372:n614. doi: 10.1136/bmj.n614. Erratum in: BMJ. 2021 Mar 19;372:n765. PMID: 33692087.
5. Samji H, Wu J, Ladak A, Vossen C, Stewart E, Dove N, Long D, Snell G. Review: Mental health impacts of the COVID-19 pandemic on children and youth – a systematic review. Child Adolesc Ment Health. 2021 Aug 28. doi: 10.1111/camh.12501. Epub ahead of print. PMID: 34455683.
6. NHS Digitial. Mental Health of Children and Young People in England, 2021: Wave 2 follow up to the 2017 survey: NHS Digital, Health and Social Care Information Centre, 2021. https://files.digital.nhs.uk/97/B09EF8/mhcyp_2021_rep.pdf
7. Hudson LD, Chapman S, Street KN, Nicholls D, Roland D, Dubicka B, Gibson F, Mathews G, Viner RM. Increased admissions to paediatric wards with a primary mental health diagnosis: results of a survey of a network of eating disorder paediatricians in England. Arch Dis Child. 2021 Sep 14:archdischild-2021-322700. doi: 10.1136/archdischild-2021-322700. Epub ahead of print. PMID: 34521633.
8. Dubicka et al. (2017). Mental health services for children fail to meet soaring demand. BMJ 2017 Sep 29;358:j4254. Doi: 10.1136/bmj.j4254.
9. Wilkinson H, Hills D, Penn A, Barbrook-Johnson P (2021) Building a system-based Theory of Change using participatory systems mapping. Evaluation 27(1) 80-101.
10. Maini R, Mounier-Jack nS, Borghi J (2018) How to and how not to develop a theory of change to evaluate a complex intervention-reflections on an experience in the Democratic Republic of Congo BMJ Global Health 3: e000617.
11. Breuer E, Lee L, DeSilva M, Lund C (2016) Using theory of change to design and evaluate public health interventions-a systematic review Implementation Science 11:63.
12. Involving parents and carers in children and young people's mental health. YoungMinds 2020. https://www.youngminds.org.uk/media/h4uexcw1/ym-parent-carer-toolkit-2020.pdf

Confidential: For Review Only

13. Gerring, J., & Cojocaru, L. (2016). Selecting Cases for Intensive Analysis: A Diversity of Goals and Methods. Sociological Methods & Research, 45(3), 392–423. https://doi.org/10.1177/0049124116631692
14. Dey I. Grounding Grounded Theory: Guidelines for Qualitative Inquiry. San Diego: Academic Press; 1999.
15. Morse, M. and Field, A. (1995) Qualitative Research Methods for Health Professionals. Sage Publications, Thousand Oaks.

AUTHOR'S CONTRIBUTIONS

LH, FG, HR and RV were major contributors to the development of the study protocol. AVV drafted the manuscript. All authors contributed to revising the manuscript and approved the final version.

FUNDING STATEMENT

This study is supported by NIHR grant number 135036.

COMPETEING INTEREST STATEMENT

None declared.

Confidential: For Review Only

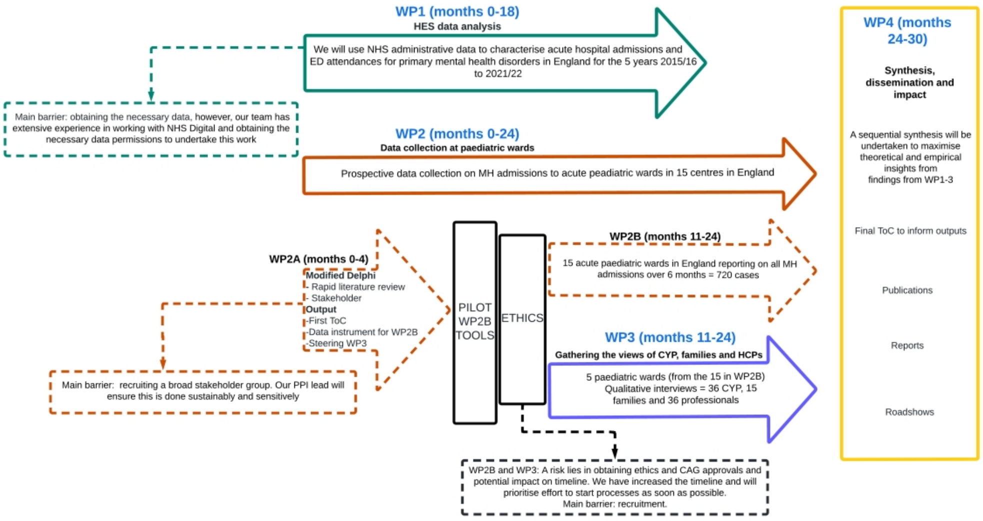

1243x670mm (118 x 118 DPI)

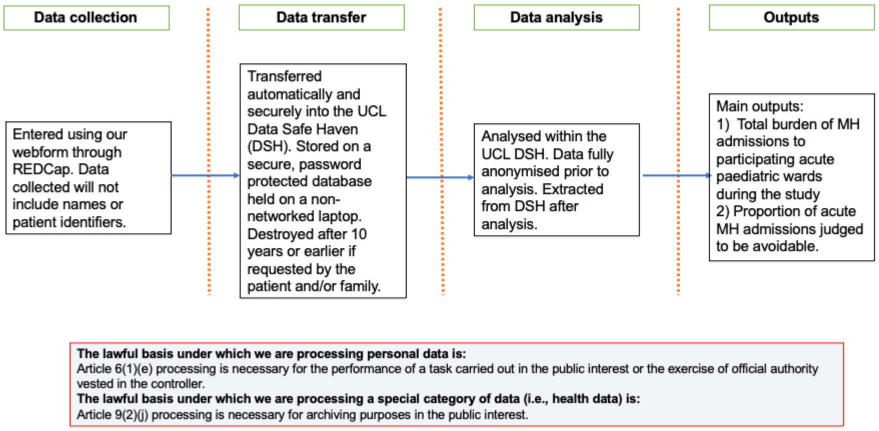

84x47mm (300 x 300 DPI)

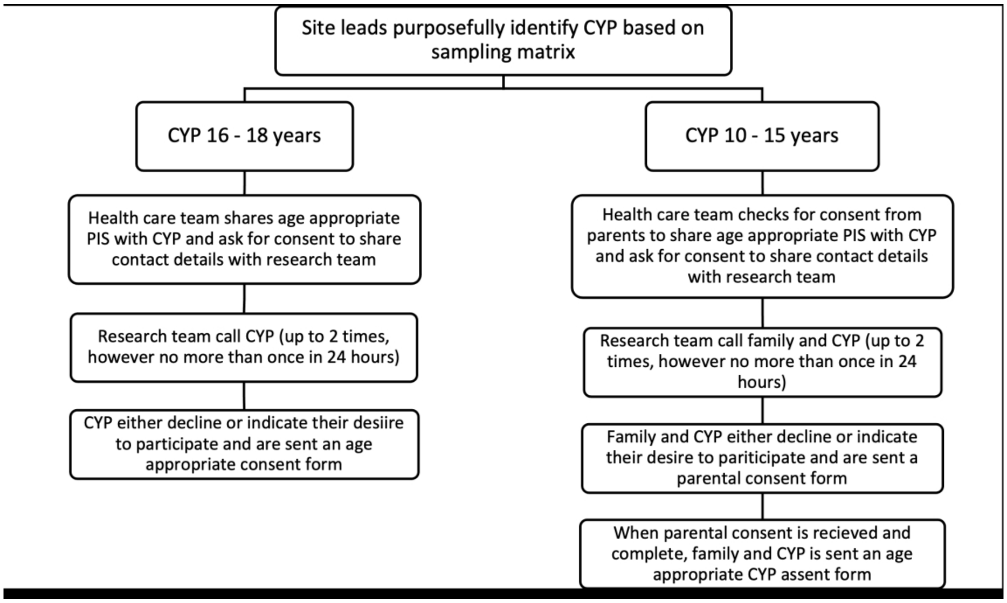

175x104mm (300 x 300 DPI)

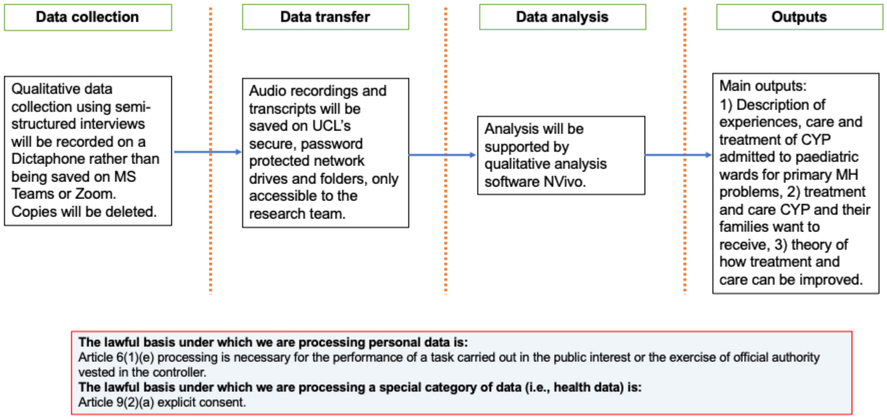

84x47mm (300 x 300 DPI)
